# Unleashing synergistic co-sensitization of BOA dyes and Ru(ii) complexes for dye-sensitized solar cells: achieving remarkable efficiency exceeding 10% through comprehensive characterization, advanced modeling, and performance analysis†

**DOI:** 10.1039/d4ra04001e

**Published:** 2024-08-14

**Authors:** Safa A. Badawy, Ehab Abdel-Latif, Walid H. Mohamed, Mohamed R. Elmorsy

**Affiliations:** a Department of Chemistry, Faculty of Science, Mansoura University El-Gomhoria Street Mansoura 35516 Egypt safabadawy140@gmail.com m.r.elmorsy@gmail.com; b Department of Chemistry, Faculty of Science, New Mansoura University New Mansoura 35712 Egypt

## Abstract

Dye-sensitized solar cells (DSSCs) have emerged as a promising alternative for renewable energy conversion. The synthesis and characterization of the 2-acetonitrile-benzoxazole (BOA) sensitizer MSW-1–4 are presented along with their chemical structures. Four new organic dyes, MSW-1 through MSW-4, were synthesized using BOA as the main building block, with different additional donor groups. The dyes were characterized and their photophysical and electrochemical properties were studied. Computational modeling using density functional theory (DFT) was performed to investigate their potential as sensitizers/co-sensitizers for photovoltaic applications. The modeling showed a distinct charge separation between the donor and acceptor parts of the molecules. For dye-sensitized solar cells, MSW-4 performed the best out of MSW-1–3 and was also better than the reference dye D-5. Moreover, MSW-3 was co-sensitized along with a typical highly efficient bipyridyl Ru(ii) sensitizer, N719, reference dye D-5, and metal-free dye MSW-4, to induce light harvesting over the expanded spectral region and hence improve the efficiency. Co-sensitizer (MSW-3 + N719) showed an improved efficiency of 10.20%. This outperformed a solar cell that used only N719 as the sensitizer, which had an efficiency of 7.50%. The appropriate combined dye loading of MSW-3 + N719 enabled good light harvesting and maximized the photoexcitation. The synergistic effect of using both MSW-3 and N719 as co-sensitizers led to enhanced solar cell performance compared with using N719 alone.

## Abstract 2

The synthesis and characterization of four 2-acetonitrile-benzoxazole (BOA) sensitizers MSW-1–4 with different additional donor groups are presented along with their chemical structures.

## Graphical abstract

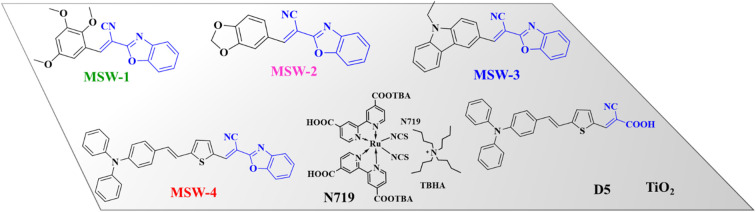

## 1 Introduction

**Fig. 1:**
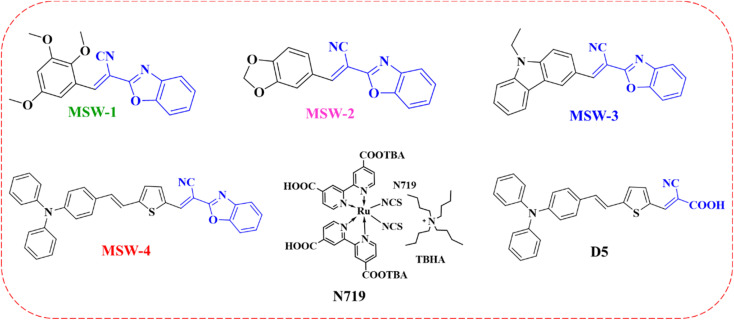
Chemical structures of the co-sensitizers MSW-1–4, D-5, and N719.

To achieve sustainable development goals, fossil fuels must no longer be considered the main source of energy. Today's photovoltaic systems do not generate greenhouse gases in comparison with fossil fuel burning, which increases the atmospheric carbon dioxide (CO_2_) concentration.^1^ Solar energy conversion is considered to be the most important route towards renewable energy. Since the Shockley–Queisser efficiency limit determined that the limit for a single-junction solar cell is approximately 33.5% under the standard AM 1.5 G, numerous studies have been conducted to achieve high efficiency, which even exceeds this limit by applying other strategies.^2^ Compared to silicon solar cells, DSSC are easy to fabricate, low-cost, and can work in dusky weather or under low-intensity light, and their overall efficiency is not affected by high temperatures.^3,4^ The dye sensitizer is the photosensitive material of the DSSC device, as it is responsible for light harvesting and generating electrons.^5,6^ Ruthenium(ii)-based sensitizers are mainly used due to their high-power conversion efficiency (PCE).^7^ The newly introduced metal-free sensitizers were ineffective due to their poor stability, narrow absorption in the visible region, and lower photovoltaic efficiency in comparison to Ru complexes.^8^ Materials with delocalized π-electron systems can absorb sunlight.^9^ Dyes are mainly synthesized with a donor–π–acceptor (D–π–A) structure, which enhances the photo-induced charge separation.^10^ The π-conjugated system has a wide range of applications, as it plays a significant role in adjusting the HOMO and LUMO energy levels and broadening the absorption range of the dye.^11^ Moreover, π-spacer modification can enhance communication between the donor and acceptor moieties, resulting in highly efficient DSSCs.^12^ The dye is also synthesized with a suitable structure and should have anchoring groups to allow it to be chemically bonded to the porous surface of the semiconductor.^13^ Ideally, the dye sensitizer should have a panchromatic effect and be able to absorb the solar spectrum from ultraviolet (UV) to near-infrared (NIR) wavelengths.^14^ This hypothesis cannot be achieved using a single dye but rather with two or more dyes as co-sensitizers in the same cell. Co-sensitizers are fabricated in DSSCs to enhance their performance and broaden their light-harvesting abilities.^15^ The dip in the absorption intensity at approximately 400 nm reflects the incident photon-to-current conversion efficiency (IPCE), resulting in a decreased PCE value. Filling this dip can be achieved using co-sensitizers, giving rise to DSSC performance.^16^ Metal-free co-sensitizers with D–π–A structure, with high molar extinction coefficient *ε*, obtain great promises as co-sensitizers for ruthenium containing DSSCs. In this study, we used 2-(benzoxazol-2-yl)-acetonitrile (BOA) as an acceptor with various donor moieties to synthesize MSW-1–4 sensitizers, as shown in Fig. 1. The study of the effect of various electron-withdrawing abilities of (BOA) on the photophysical and electronic properties and photovoltaic performance of MSW-1–4 was carried out in detail. Therefore, to further investigate the photovoltaic properties of MSW-3, we incorporated novel dyes with N719,^17^ the reference dye D-5.^18^

## 2 Experimental section

### 2.1. Materials and methods

The ESI† includes a comprehensive list of the equipment, tools, and compounds employed to characterize and synthesize the dyes and fabricate DSSCs.

### 2.2. Synthesis

#### 2.2.1. General method for the synthesis of 2-acetonitrile-benzoxazole (BOA) sensitizer MSW-1–4

In a conical flask, 2-(benzo[*d*]oxazol-2-yl)acetonitrile (BOA) 1 (1.58 g, 0.01 mol) was added to 3,4,5-trimethoxybenzaldehyde 2 (1.96 g, 0.01 mol), benzo[*d*][1,3]dioxole-5-carbaldehyde 3 (1.50 g, 0.01 mol), 9-ethyl-9*H*-carbazole-3-carbaldehyde 4 (3.63, 0.01 mol) in 100 mL absolute ethanol, drops of piperidine as a basic catalyst, and finally 5-(4-(diphenylamino)styryl)thiophene-2-carbaldehyde 10 (3.81, 0.01 mol) in 100 mL acetonitrile (MeCN) and drops of piperidine as a basic catalyst; the reaction mixtures were refluxed for 8 h. The sensitizers formed (MSW-1–4) were filtered and washed with ethanol.

##### 2.2.1.1. 2-(Benzo[*d*]oxazol-2-yl)-3-(2,3,5-trimethoxyphenyl)acrylonitrile (MSW-1)

Yellow sheets (75% yield) mp = 274–276 °C. IR (*ν̄*, cm^−1^): 2965, 2931 (CH), 2212 (CN), 1617 (C=C). ^1^H NMR (DMSO-*d*_6_, ppm): *δ* 3.79 (s, 3H, OCH_3_), 3.94 (s, 6H, OCH_3_), 6.84 (s, 1H, Ar–H), 7.14 (s, 1H, Ar–H), 7.50–7.55 (m, 4H, Ar–H), 7.96 (s, 1H, =CH). ^13^C NMR (*δ* ppm^−1^): 56.79, 60.65 (2C), 103.34, 107.41 (2C), 109.54, 111.23, 118.25, 124.37, 125.49, 129.71, 136.67, 138.33 (2C), 141.85, 152.06, 154.29, 154.64. Analysis calcd for C_19_H_16_N_2_O_4_ (336.35): C, 67.85; H, 4.80; N, 8.33%. Found: C, 68.06; H, 4.90; N, 8.18%.

##### 2.2.1.2. 3-(Benzo[*d*][1,3]dioxol-5-yl)-2-(benzo[*d*]oxazol-2-yl)acrylonitrile (MWS-2)

Orange sheet (68% yield), mp = 210–212 °C. IR (*ν̄*, cm^−1^): 2924 (CH), 2210 (CN), 1620 (C=C). ^1^H NMR (DMSO-*d*_6_, ppm): 6.17 (s, 2H, CH_2_), 7.22 (d, 2,H, *J* = 8.50 Hz, Ar–H), 7.13 (d, 1H, *J* = 8.50 Hz, Ar–H), 7.40 (d, 2H, *J* = 9.00 Hz, Ar–H), 7.51 (d, 1H, *J* = 9.00 Hz, Ar–H), 7.16 (s, 1H, Ar–H), 8.10 (s, 1H, =CH). ^13^C NMR (*δ* ppm^−1^): 102.12, 103.71, 109.65, 110.79, 111.23, 117.26, 118.25, 123.93, 124.37, 125.49, 128.06, 137.41, 138.33, 148.77, 150.09, 152.06, 152.36. Analysis calcd for C_17_H_10_N_2_O_3_ (290.28): C, 70.34; H, 3.47; N, 9.65%. Found: C, 70.04; H, 3.35; N, 9.83%.

##### 2.2.1.3. 2-(Benzo[*d*]oxazol-2-yl)-3-(9-ethyl-9*H*-carbazol-3-yl)acrylonitrile (MWS-3)

Red crystals (89% yield), mp = 234–236 °C. IR (*ν̄*, cm^−1^): 2983, 2935 (CH), 2203 (CN), 1598 (C=C). ^1^H NMR (DMSO-*d*_6_, ppm): *δ* 1.34 (t, 3H, *J* = 7.00 Hz, CH_3_), 4.50 (q, 2H, *J* = 7.00 Hz, CH_2_), 6.73–6.77 (m, 3H, Ar–H), 7.30 (t, 2H, *J* = 7.50 Hz, Ar–H), 7.42–7.48 (m, 3H, Ar–H), 7.70 (d, 1H, *J* = 8.00 Hz, Ar–H), 7.82 (d, 1H, *J* = 8.00 Hz, Ar–H), 8.41 (s, 1H, =CH), 8.78 (s, 1H, Ar–H). ^13^C NMR (*δ* ppm^−1^): 13.33, 42.69, 103.71, 105.06, 109.08, 109.54, 111.23, 118.25, 123.99, 124.37, 125.49, 126.09, 129.10, 137.41, 138.33, 139.73, 141.74, 152.06, 154.64. Analysis calcd for C_24_H_17_N_3_O (363.42): C, 79.32; H, 4.72; N, 11.56; found: C, 79.02; H, 4.62; N, 11.44%.

##### 2.2.1.4. 3-(5-(4-(Diphenylamino)styryl)thiophen-2-yl)-2-cyanoacrylic acid (D-5)

The synthetic procedure and characterization have been presented in an earlier publication.^18^

##### 2.2.1.5. 2-(Benzo[*d*]oxazol-2-yl)-3-(5-((*E*)-4-(diphenylamino)styryl)thiophen-2-yl)acrylonitrile (MSW-4)

Dark-red crystals (75% yield), mp = 250–252 °C. IR (*ν̄*, cm^−1^): 2902, 2819 (CH), 2209 (CN), 1612 (C=C). ^1^H NMR (DMSO-*d*_6_, ppm): *δ* 6.92 (t, 2H, *J* = 7.00 Hz, CH_3_), 7.02 (d, 2H, *J* = 8.00 Hz, Ar–H), 7.04 (d, 2H, *J* = 4.00 Hz, H–thiophene), 7.18–7.20 (m, 3H, Ar–H), 7.23 (d, 2H, *J* = 8.00 Hz, Ar–H), 7.26 (d, 2H, *J* = 12.00 Hz, C=CH), 7.30–7.34 (m, 5H, Ar–H), 7.41–7.44 (m, 2H, Ar–H), 7.48 (d, 2H, *J* = 8.00 Hz, Ar–H), 7.53 (s, 1H, C=CH). ^13^C NMR (*δ* ppm^−1^): 98.80, 111.23, 111.76, 118.25, 124.37, 124.88 (2C), 125.49, 125.91, 126.27 (4C), 126.62 (2C), 127.82, 129.47, 129.80 (4C), 131.16 (2C), 133.36 (2C), 137.75, 138.33, 138.73, 139.47, 140.31, 145.43 (2C), 151.82, 152.06. Analysis calcd for C_34_H_23_N_3_OS (521.64): C, 78.29; H, 4.44; N, 8.06%. Found: C, 78.06; H, 4.59; N, 7.86%.

## 3 Results and discussion

### 3.1. Synthesis and structural characterization

**Scheme 1:**
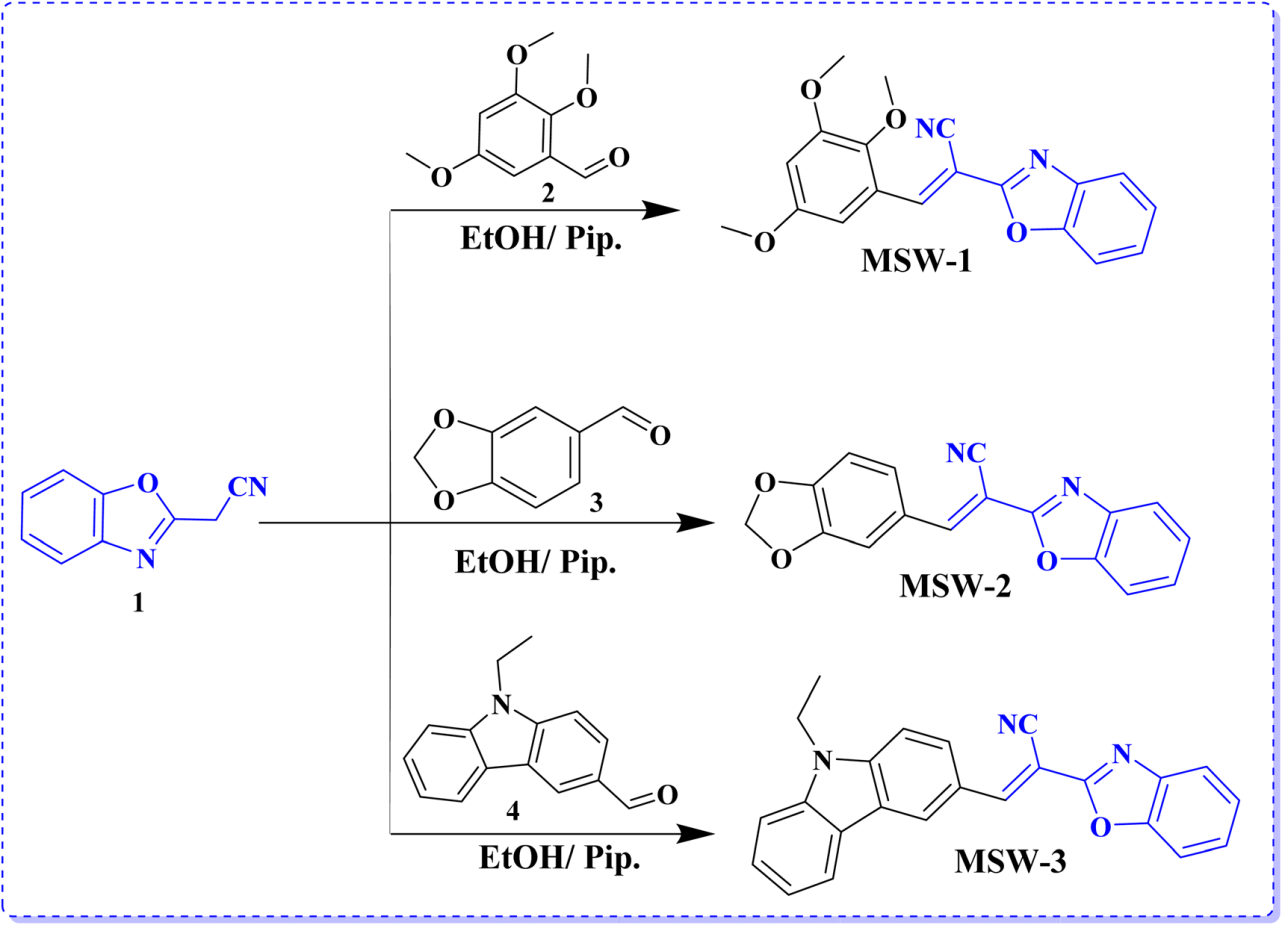
Synthesis of benzoxalylacetonitrile sensitizer MSW-1–3.

MSW-based dyes have received particular attention as representative acceptors. 2-(Benzo[*d*]oxazol-2-yl)acetonitrile (BOA) has an electron-withdrawing ability due to the intrinsic electron-deficient character of both benzoxazole and cyano groups. The reaction of 3-(3,5-dimethyl-1*H*-pyrazol-1-yl)-3-oxopropanenitrile with 2-aminophenol in refluxing toluene results in the formation of benzoxazol-2-yl-acetonitrile (BOA) (1), as shown in Scheme 1.^19^ The sensitizer MSW-1–4 was formed in high yield through the Knoevenagel condensation of 2-(benzo[*d*]oxazol-2-yl)acetonitrile (1) with various donor aldehydes such as 2,3,5-trimethoxybenzaldehyde (2), benzo[*d*][1,3]dioxole-5-carbaldehyde (3), and 9-ethyl-9*H*-carbazole-3-carbaldehyde (4) in ethanol and drops of piperidine to afford the oxazolylacetonitrile sensitizer MSW-1–3 (Scheme 1) in good yield. The structures of the newly synthesized sensitizers, MSW-1–3, were confirmed using various spectroscopic techniques. The IR spectrum of MSW-1 showed aliphatic (CH) groups at 2965 and 2931 cm^−1^, in addition to a broad absorption at 2212 cm^−1^ for the cyano group (CN). The ^1^H NMR spectrum of MSW-1 showed a nine singlet signals at *δ* 3.79 and 3.94 ppm related to three methoxy group (OCH_3_). The vinylic proton (C=CH) resonated as a singlet at *δ* 7.69 ppm. The ^13^C NMR spectrum of MSW-1 exhibited signals for aliphatic carbon atoms in the expected regions of 56.79 (OCH_3_) and 60.65 (2OCH_3_). In addition, the signals for aromatic carbon atoms were indicated by their chemical shifts: 103.34, 107.41 (2C), 109.54, 111.23, and 118.25, 124.37, 125.49, 129.71, 136.67, 138.33 (2C), 141.85, 152.06, 154.29, 154.64 ppm. The IR spectrum of MSW-2 showed a strong absorption at 2210 cm^−1^ for the cyano group. The ^1^H NMR spectrum showed two singlet signals at *δ* 6.17 and 8.10 ppm related to methylene (CH_2_) and vinylic proton (C=CH). The ^1^H NMR spectroscopic analysis of MSW-3 revealed distinctive signals characteristic of its molecular structure. The ethyl group attached to the carbazole moiety exhibited a characteristic splitting pattern. Specifically, the methyl protons (CH_3_) appeared as a triplet at a chemical shift of *δ* 1.34 ppm. The adjacent methylene protons (CH_2_) manifested as a quartet at *δ* 4.50 ppm. Additionally, the spectrum showed a notable singlet at *δ* 8.41 ppm, which can be attributed to the vinylic proton (C=CH).

**Scheme 2:**
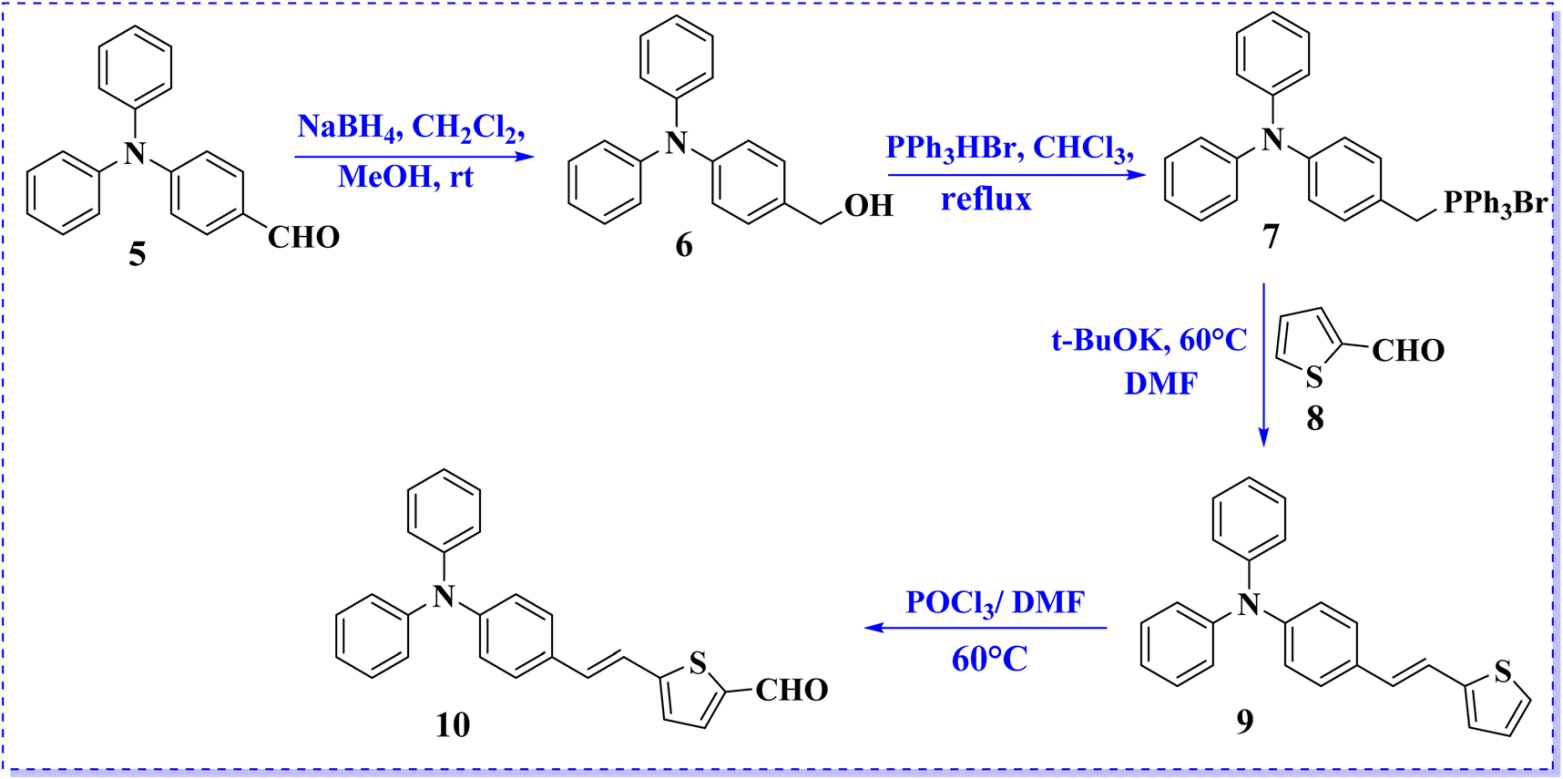
Synthesis of precursor aldehyde (10).

Scheme 2 depicts the synthetic route for the precursor aldehyde (10), which involves Wittig reaction of triphenylphosphine salt (7) with 2-formylthiophene (8) to generate the corresponding thiophene compound (9). Subsequently, intermediate 9 was formylated using the Vilsmeier–Haack protocol to afford thiophene-2-carbaldehyde derivative (10) in satisfactory yield. The melting points and spectroscopic properties of all compounds were consistent with those reported in the literature.^20^

**Scheme 3:**
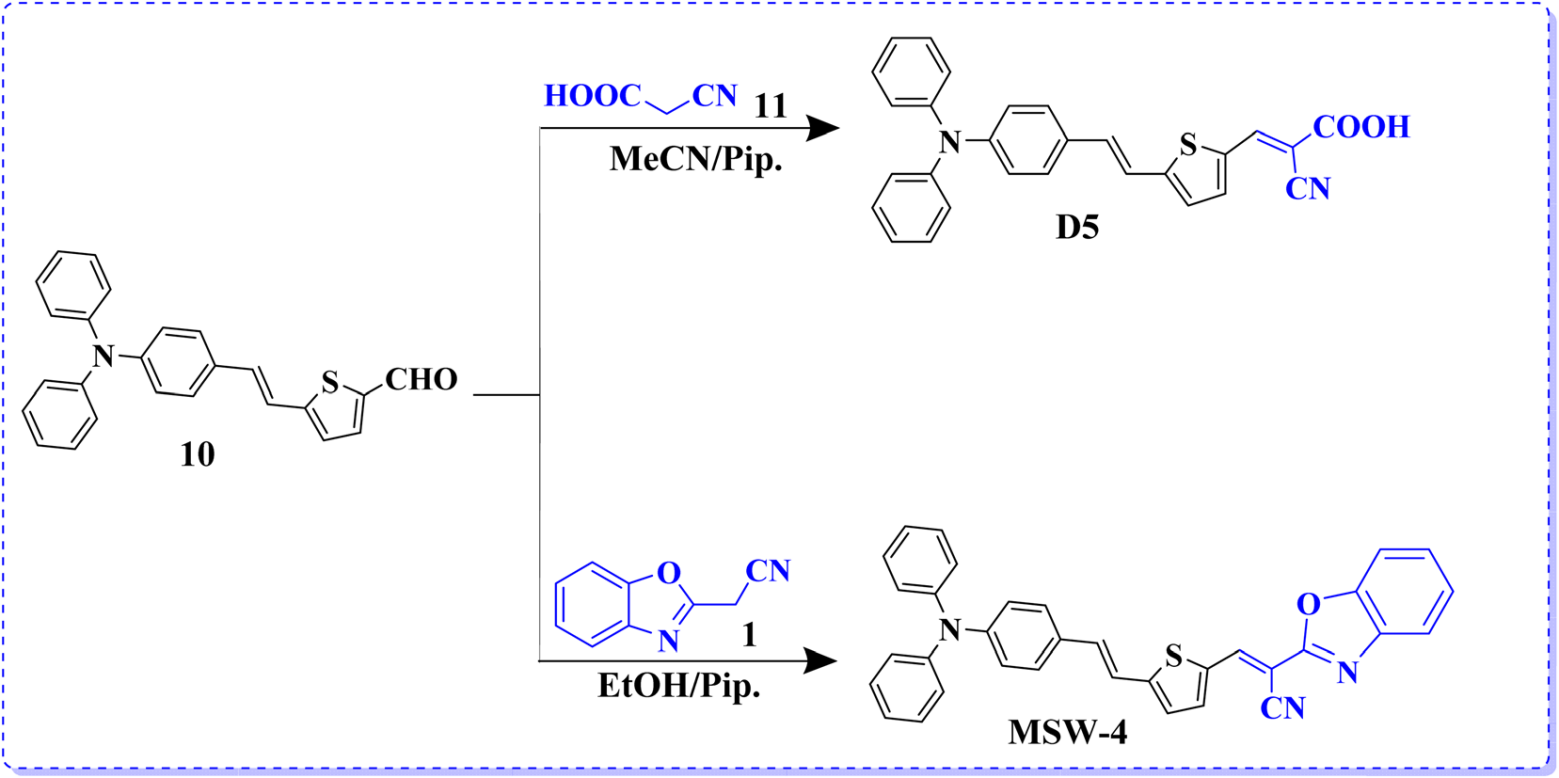
Synthesis of benzoxalylacetonitrile sensitizers, MSW-4 and D-5.

Sensitizer D-5 was synthesized *via* Knoevenagel condensation, involving the condensation of 5-(4-(diphenylamino) styryl)thiophene-2-carbaldehyde (10) with cyanoacetic acid (11) in the presence of piperidine and acetonitrile (MeCN) solution refluxed for 6 h. A previously reported melting point of the synthesized product was observed.^18^ Finally, the end sensitizer MSW-4 was formed in high yield through the Knoevenagel condensation of 5-(4-(diphenylamino) styryl)thiophene-2-carbaldehyde (10) with benzoxazol-2-yl-acetonitrile (BOA) (1) to afford dark-red crystals in good yield. The infrared (IR) spectrum of MSW-4 displayed characteristic absorption bands for the cyano group (C≡N) at 2209 cm^−1^, as well as stretching vibration bands of the vinyl groups at 1612 cm^−1^. Additionally, the ^1^H NMR spectrum of MSW-4 exhibited a doublet signal at *δ* 7.26 ppm, attributed to the two protons of the vinylic group (*J* = 12.00 Hz) and two protons of thiophene, which also appeared as doublet signals at *δ* 7.04 ppm (Scheme 3).

### 3.2. Optical properties of benzoxalylacetonitrile co-sensitizer MSW-1–4

**Fig. 2:**
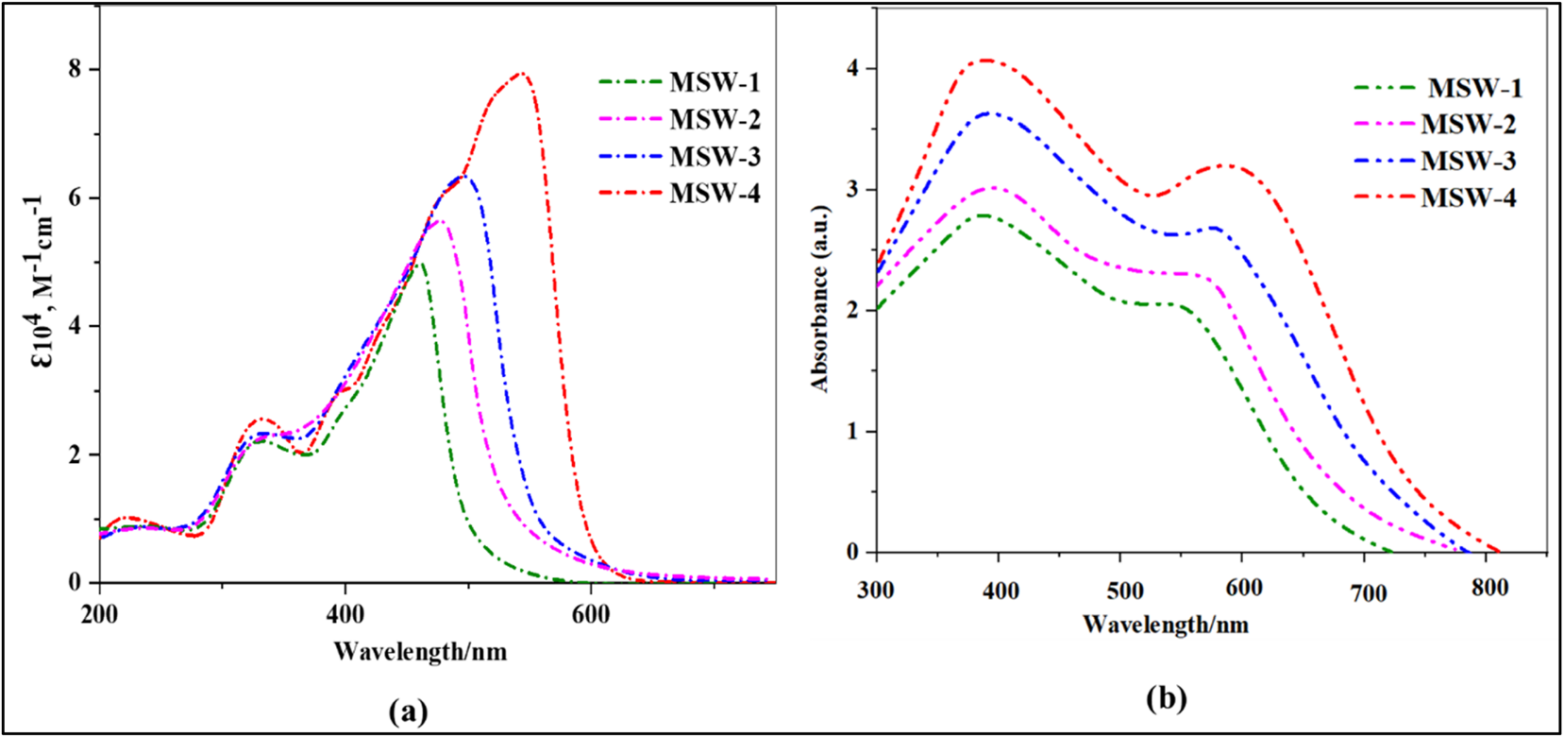
(a) UV-Vis absorption of benzoxalylacetonitrile (BOA) sensitizer MSW-1–4 in solution, (b) Absorption of the sensitizer MSW-1–4 on TiO_2_.

Fig. 2a displays the essential UV-visible absorption spectrum properties of the oxazolylacetonitrile sensitizer MSW-1–4 dissolved in DMF at a concentration of 2 × 10^−5^ M. Furthermore, Table 1 presents a comprehensive collection of pertinent facts about MSW-1–4 sensitizers.

**Table 1:** Optical parameters of BOA sensitizer MSW-1–4

Sensitizer	*λ*_max_ (nm)	*ε* (10^4^ M^−1^ cm^−1^)	*λ*_onset_/nm	Experimental *E*_0–0_ (eV)
MSW-1	(321, 460)	(2.16, 5.09)	500	2.47
MSW-2	(324, 480)	(2.33, 5.68)	550	2.25
MSW-3	(322, 495)	(2.35, 6.34)	578	2.14
MSW-4	(325, 546)	(2.62, 7.92)	612	2.02

In a broad sense, all five structures exhibited absorption bands ranging from 220 to 400 nm, which aligned with the connected aromatic π–π* transition. Strong peaks were observed in the low-energy zone, that is, in the wavelength range-420–620 nm.^21^ These peaks may be attributed to an intramolecular charge transfer (ICT) shift occurring across the donor and acceptor groups. According to the data shown in Fig. 2a, the maximum wavelengths (*λ*_max_) for MSW-1–4 compounds in the ICT process were 460, 480, 495, and 546 nm, respectively. (Benzoxalylacetonitrile) BOA has an electron-withdrawing ability due to character of both benzoxazole and cyano groups.^22^ Furthermore, the associated molar extinction coefficient (*ε*) values for these compounds are determined to be 5.09, 5.68, 6.34, and 7.92 M^−1^ cm^−1^, correspondingly. In other words, the order of increase is MSW-4 > MSW-3 > MSW-2 > MSW-1, as shown in Table 1. The bandgap can also be determined by analyzing the onset of the UV-visible absorption spectra (*E*_0–0_).^23^ The user provided a numerical reference. The determined *E*_0–0_ energies for the dyes were 2.47, 2.25, 2.14 eV, and 2.02 eV. It is evident that MSW-4 exhibits a red-shifted *λ*_max_ and possesses higher molar absorptivity than MSW-1–3. The enhanced visible light absorption of the sensitizer MSW-4 can be attributed to the addition of a thiophene π-bridge group, which was enhanced by energy delocalization and increased polarizability. This, along with the strong triphenylamine donor group and the effective push–pull system between the donor and acceptor, improved light harvesting, sensitizer MSW-3 displayed a red shift than MSW-1–2. This can be explained by the presence of carbazole, which is a strong electron-donating group, along with a benzoxazolylacetonitrile acceptor. The carbazole donor and synergistic push–pull system resulted in the red-shifted absorption of MSW-3. Fig. 2b illustrates the absorption spectra of the dyes when attached to TiO_2_ films. A comparison between these spectra and those of sensitizers in solution reveals a notable bathochromic shift for MSW-1–4 sensitizers. This red shift can be attributed to the formation of J-aggregates.^24,25^ The formation of coordinate bonds between the nitrogen atom in the oxazolylacetonitrile of the dyes and the Lewis acid sites of the TiO_2_ surface was demonstrated to result in efficient electron injection. This work suggested that the first use of the benzoxazole ring acted not only as an electron-withdrawing anchoring group but also as an electron-injecting group. The dye loading on the TiO_2_ surface decreased in the order MSW-4 > MSW-3 > MSW-2 > MSW-1. Thus, introducing a triphenylamine unit and incorporating a thiophene moiety and achieve a greater loading capacity on the TiO_2_ layer than MSW-1–3.^26^ However, the absorption intensity of MSW-4 based film was stronger than that of MSW-1–3, which indicates that the amount of MSW-4 adsorbed on TiO_2_ may be greater than that of other sensitizers. Consequently, MSW-4 is expected to improve the performance of DSSCs in terms of its broadest light response to TiO_2_. This attribute is advantageous for achieving high *J*_SC_ values and enhanced light-harvesting capabilities.

### 3.3. Quantum chemical parameter for BOA sensitizers MWS-1–4

To investigate the molecular structures of the MSW-1–4 sensitizers, we employed density functional theory (DFT) calculations. These computations were carried out using the Gaussian 09 software package.^27^ The chemical reactivity of the synthesized sensitizers was assessed by quantum analysis within the framework of Koopmans' hypothesis. This analysis involves the computation of several crucial parameters, such as ionization energy (IP), electron affinity (EA), hardness (*η*), softness (*s*), electronegativity (*χ*), and chemical potential (*μ*),^28^Table 2 displays the relevant data, and the calculations were conducted in accordance with eqn (1)–(5).

1 IP = −*E*_HOMO_

2 EA = −*E*_LUMO_

3
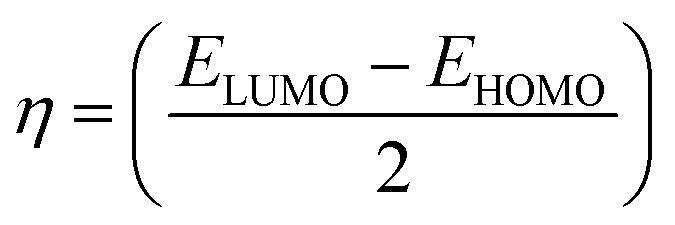

4 *s* = (1/*η*)

5 *χ* = −*μ* = −(*E*_LUMO_ + *E*_HOMO_)/2

**Table 2:** Quantum chemical parameters of benzoxalylacetonitrile BOA sensitizer MSW-1–4

Theoretical	MSW-1	MSW-2	MSW-3	MSW-4
HOMO (eV)	−5.92	−5.64	−5.60	−5.30
LUMO (eV)	−3.45	−3.41	−3.45	−3.29
*E*_0–0_ (eV)	2.47	2.23	2.15	2.01
IP (eV)	5.92	5.64	5.60	5.30
EA (eV)	3.45	3.41	3.45	3.29
*η* (eV)	1.23	1.11	1.07	1.00
*s* (eV)	0.81	0.90	0.93	1.00
*μ* (eV)	−4.68	−4.52	−4.52	−4.29
*χ* (eV)	4.68	4.52	4.52	4.29

IP values of the investigated molecules were in the following order: MSW-1 (5.92 eV) > MSW-2 (5.64 eV) > MSW-3 (5.60 eV) > MSW-4 (5.30 eV). The results obtained from the measurement of the IP values revealed that MSW-4 exhibited the lowest values, indicating its efficiency in effective charge transfer. The range of EA values for the investigated molecule (3.29–3.45 eV) confirmed the superior electron transport performance of MSW-4, which is attributed to the strength of its donor group (TPA) and the presence of various acceptor moieties. An inverse correlation between global hardness and reactivity was demonstrated. As the global hardness decreased, the reactivity increased.^29^ It has been reported that the global hardness for sensitizers (MSW-1–4) was found to be MSW-1 (1.23 eV) > MSW-2 (1.11 eV) > MSW-3 (1.07 eV) > MSW-4 (1.00 eV) respectively. The electronegativity of the sensitizers ranged from 4.29 to 4.68 eV. These results suggest that the porphyrin sensitizer MSW-1–4 undergoes intramolecular charge transfer more easily because of the interaction between its π-system and acceptor groups. Sensitizer MSW-4 displayed a conjunction of low (IP), high (EA), and low *η*, which resulted in augmented intramolecular charge transfer and elevated (*J*_SC_) values.

### 3.4. Molecular modeling of BOA sensitizer MSW-1–4

**Fig. 3:**
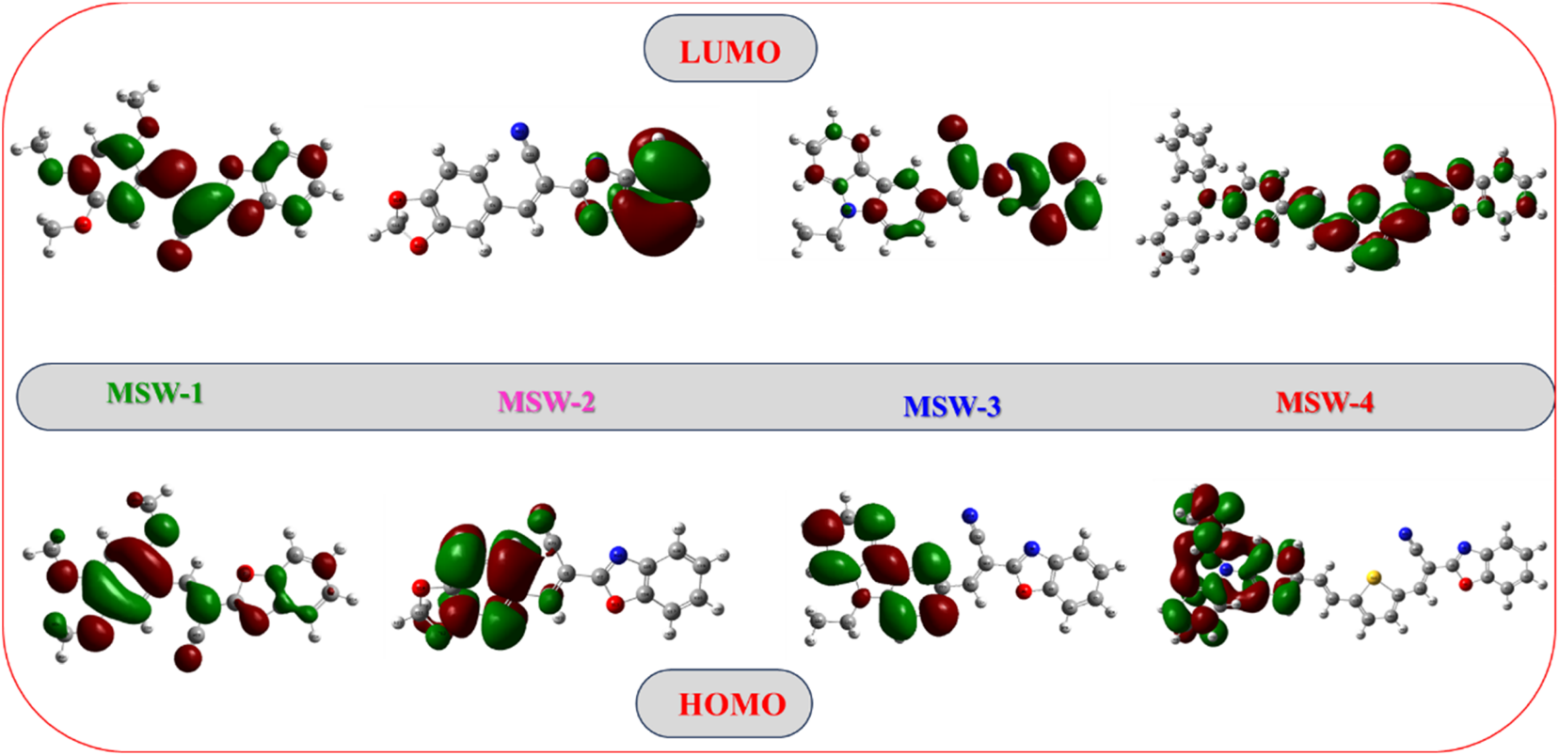
FMO for the benzoxalylacetonitrile sensitizer, MSW-1–4.

Intramolecular charge transfer (ICT) plays a pivotal role in the performance of sensitizers (MSW-1–4).^30^Fig. 3 illustrates the electron density distributions of the MSW-1–4 dyes, revealing distinctive patterns across the series. For MSW-1, the highest occupied molecular orbitals (HOMO) spread evenly across the entire molecule. In contrast, the lowest unoccupied molecular orbitals (LUMO) concentrate primarily on the acceptor region, particularly the benzoxalylacetonitrile segment. This deficient donor–acceptor electron transfer could potentially impact electrons injection and efficiency. MSW-2 and MSW-3 exhibit HOMOs predominantly localized on the piperonal and ethylcarbazole groups, respectively. A π-bridge facilitates electron density diffusion to acceptor units like oxazolylacetonitrile. The LUMOs of these dyes show significant delocalization across various oxazolylacetonitrile acceptors. This delocalization is crucial for enabling effective electronic coupling between the dye and TiO_2_ surface, facilitating efficient electron transfer from excited dyes to the TiO_2_ conduction band.^30^ In the MSW-4 sensitizer, HOMO electron density localizes primarily on the donor (triphenylamine) and extends to the π-bridge (thiophene ring). Conversely, LUMO electron density concentrates on the π-bridge and the novel oxazolylacetonitrile acceptor.

### 3.5. Molecular electrostatic potential (MEP) of sensitizers, electron localization function (ELF), and localized orbital locator (LOL) analysis of MSW-1–4

**Fig. 4:**
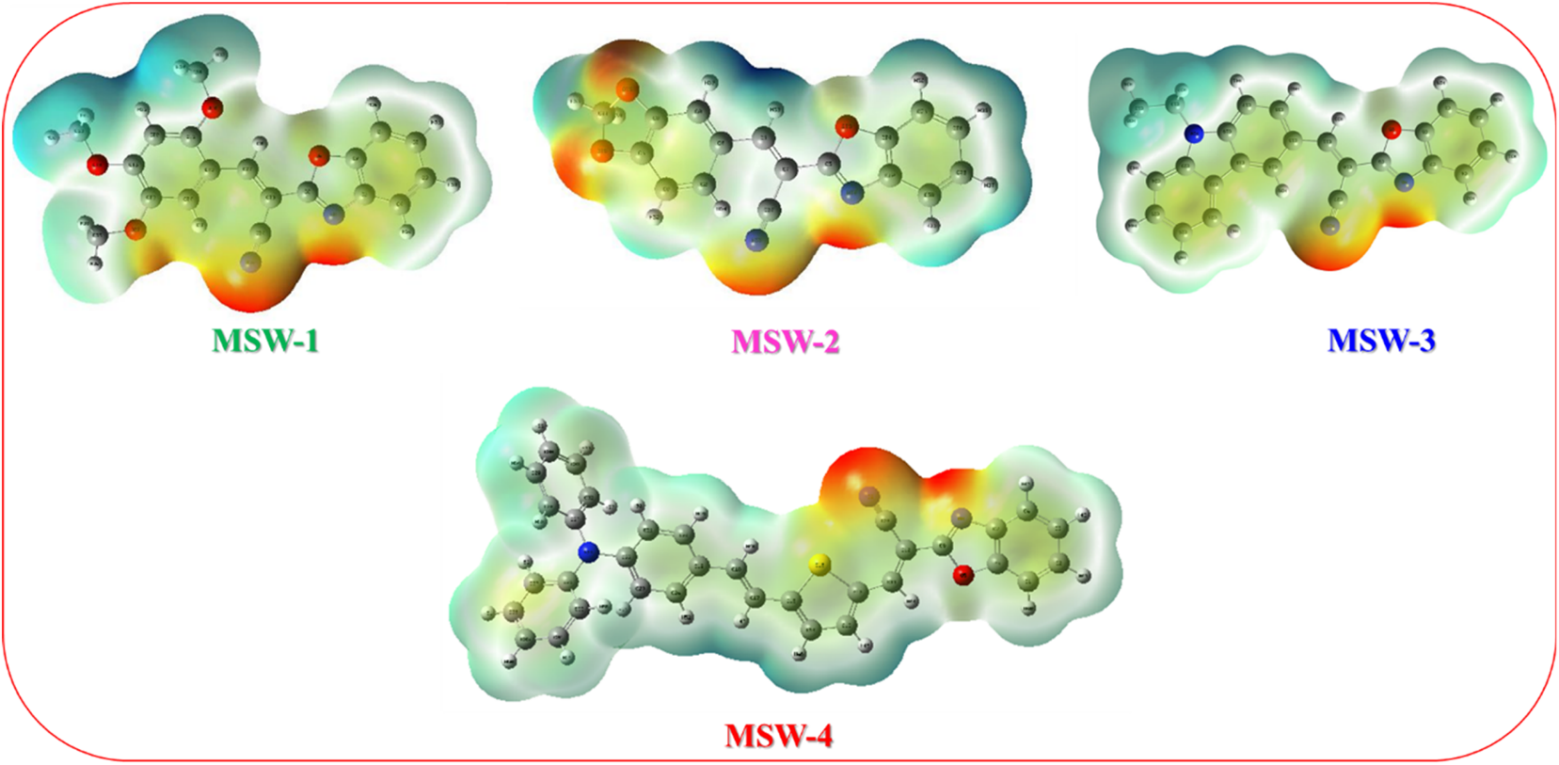
MEP maps of the co-sensitizer oxazolylacetonitrile sensitizer MSW-1–4.

MEP analysis provides a visual representation of the charge distribution across a molecule's surface, offering crucial insights into its chemical reactivity.^31^ The MEP map depicts repulsive nuclei as positively charged regions (shown in blue) and attractive electron interactions as negatively charged regions (shown in yellow and red) as shown in Fig. 4. It is noteworthy that for the oxazolylacetonitrile sensitizer MSW-1–4, the negative regions (red) are around the oxazolylacetonitrile segment of the electron-withdrawing groups of benzoxalylacetonitrile (BOA). This observation indicates their electrophilic reactivity. In contrast, the positive (blue) areas in the sensitizer MSW-1–3 are predominantly located around the donor groups, including trimethoxy, piperonal, and *N*-ethylcarbazole moieties, and in the case of MSW-4, they are concentrated on triphenylamine extended to the thiophene moiety.

**Fig. 5:**
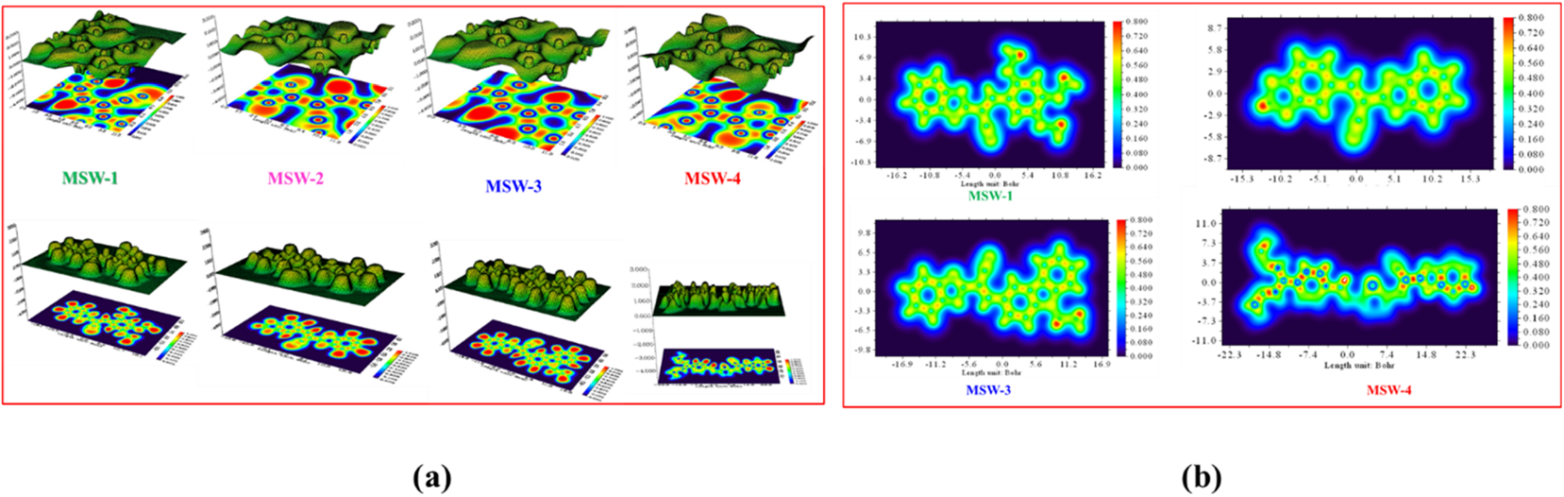
(a) ELF maps of the co-sensitizer benzoxalylacetonitrile sensitizer MSW-1–4, (b) localized orbital locator maps (LOL) for the optimized geometry of the co-sensitizer oxazolylacetonitrile MSW-1–4.

The Electron Localization Function (ELF), based on molecular surface density kinetic energy, provides insights into charge-shift bonds, chemical bonding, and shell structure.^32,33^ Introduced by Silvi and Savin, ELF analysis derives from electron pair density, with values of 0.5–1.0 indicating higher electron localization. Fig. 5a shows ELF maps for MSW-1–4 sensitizers. Electrons are predominantly localized at donor units (trimethoxy, piperonal, ethyl carbazole and triphenylamine), while the acceptor benzoxalylacetonitrile unit shows moderate localization. ELF analysis offers information on charge-shift bonds, bonding classification, and shell structure.^34^ The Localized Orbital Locator (LOL) analysis in Fig. 5b reflects overall electron distribution. It reveals symmetric electron distribution in MSW-1–4 sensitizers, crucial for maintaining their planar structure. LOL confirms higher electron localization at donor units and lower localization at acceptor oxazolylacetonitrile segments. Together, ELF and LOL analyses provide comprehensive insights into the electronic structure, bonding characteristics, and charge distribution of MSW-1–4 sensitizers, informing their potential applications in fields like dye-sensitized solar cells or organic electronics.

### 3.6. Electrochemical characterization of BOA sensitizer MSW-1–4

**Fig. 6:**
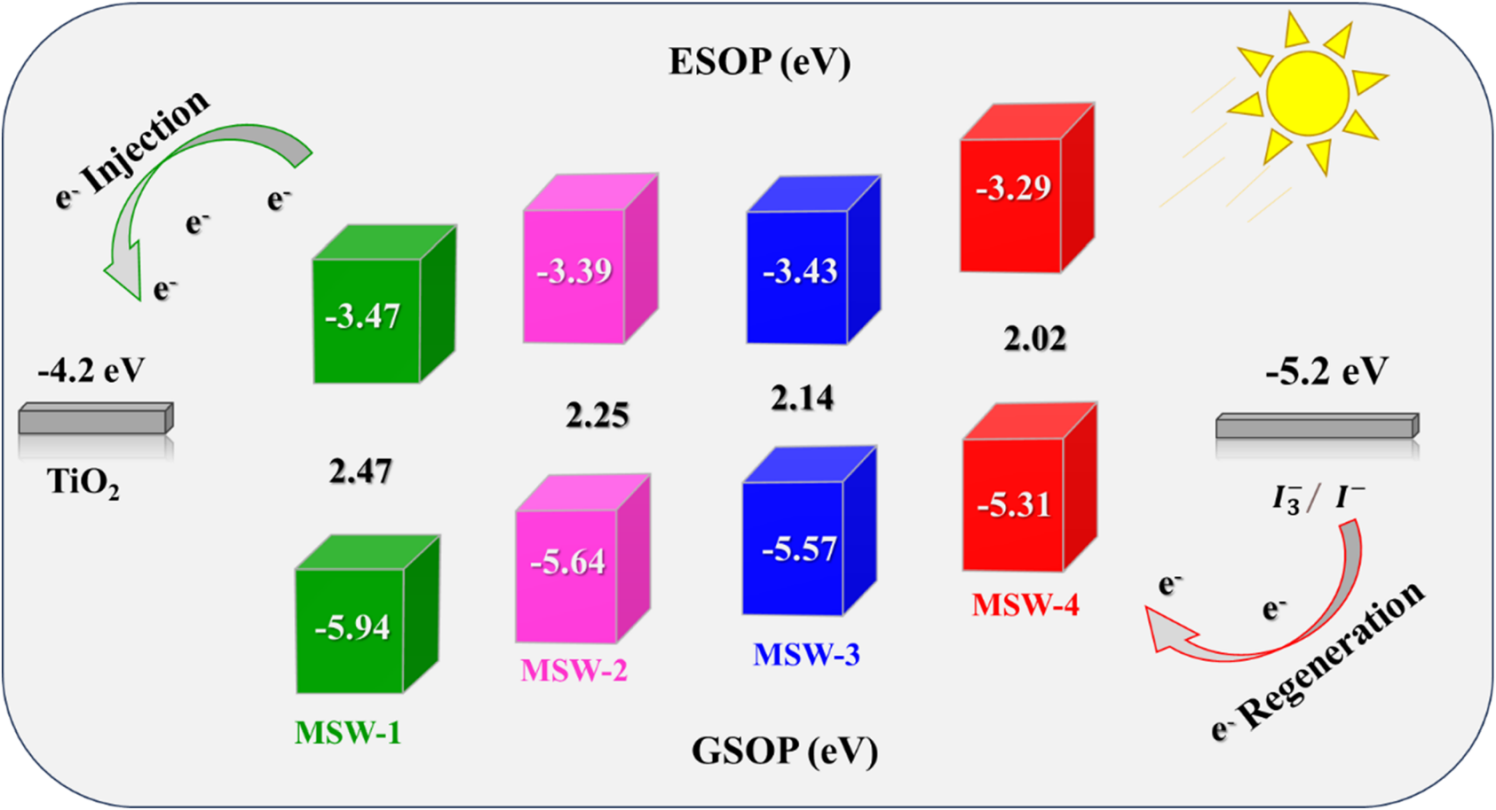
Energy level diagram of the benzoxalylacetonitrile sensitizer MSW-1–4.

CV was used to determine the oxidation potentials of the co-sensitizers MSW-1–4 in their highest occupied molecular orbital (GSOP/HOMO) and lowest occupied molecular orbital (ESOP/LUMO). The optoelectronic characteristics of these materials can be enhanced by analyzing their electrical dispersion, as shown in Fig. 6.^35^ The determination of the charge regeneration and electron implantation is significantly influenced by the HOMO and LUMO energy levels of the compound. To enhance the efficacy of introducing electrons into TiO_2_, it is essential for the compound to possess an LUMO level that surpasses the conduction band (CB) of TiO_2_. In addition, it is necessary for the sensitizer (HOMO) level to be higher than that of the electrolyte to facilitate effective dye regeneration.^35^ Hence, the energy levels of MSW-1–4 were determined and are displayed in Fig. 6, with the corresponding calculation data for comparative analysis. The GSOP/HOMO energy level was determined using eqn (6) and (7).

6 GSOP = −[oxidation onset + 4.7] eV

7 ESOP = [GSOP − *E*_0–0_] eV

The HOMO levels of the five dyes exhibit a range spanning between (−5.31 and −5.94 eV), which is comparatively negatively higher than the redox potential of the *I*_3_^−^/*I*^−^ electrolyte (−5.20 eV). This discrepancy in energy levels ensures the effective renewal of the oxidized dyes. The LUMO values of all dyes exhibit a range spanning from −3.29 to −3.47 eV, surpassing the energy level of the CB of TiO_2_. This observation suggests that the dyes possess sufficient driving power to facilitate the injection of electrons into TiO_2_. Furthermore, the optical band gap's energies (*E*_0–0_) were determined by analyzing the absorption onset, resulting in estimated values of 2.47, 2.25, 2.14, and 2.02 eV for the co-sensitizers MSW-1–4, respectively. The incorporation of a thiophene moiety resulted in the elevation of (LUMO) energy levels of MSW-4 to −3.29 eV, respectively.^35^

## 4 Photovoltaic device characterizations

**Fig. 7:**
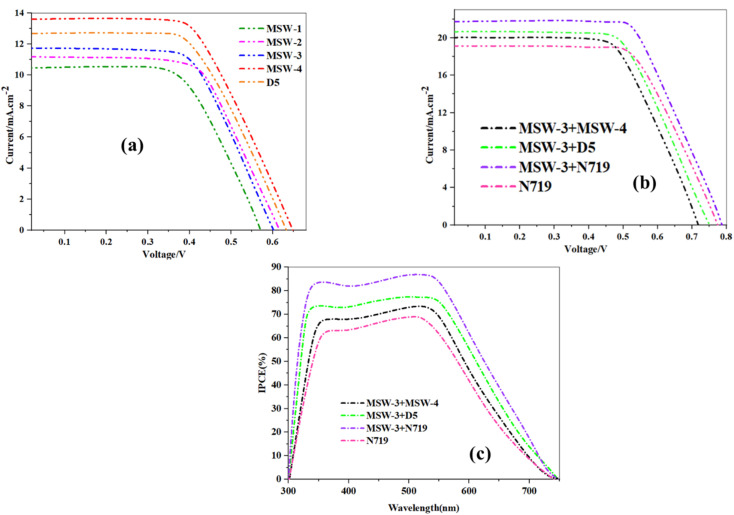
(a) *J*–*V* curves of benzoxalylacetonitrile sensitizer MSW-1–4 devices, (b) *J*–*V* curves of MSW-3–4, D-5, and N719, (c) IPCE spectra of MSW-3–4, D-5, MSW-4 and N719.

The photovoltaic measurements are shown in Table 3, and the current–voltage (*I*–*V*) characteristics of the corresponding sensitized devices using MSW-1–4 are shown in Fig. 7a. Upon sensitization, MSW-1 bearing trimethoxybenzene as a donor demonstrated the lowest efficiency an efficiency (*η*) of 3.13%. The piperonal sensitizer MSW-2 showed an efficiency with a notable value of 3.82%. It also demonstrated a (*J*_SC_) of 11.17 mA cm^−2^, (*V*_OC_) of 0.625 V, and (FF) of 54.85. The improved solar efficiency of these sensitizers may be ascribed to the variety of donor moieties and elevated levels of both photocurrent and photovoltage. The highest values of *J*_SC_ for MSW-3, MSW-4, comparing to MSW-1–2 reached to (11.65 and 13.60 mA cm^−2^) which indicate their ability to effectively capture light, incorporate dyes, and achieve energy alignment within the DSSC framework.^36,37^ Moreover, the rationale behind MSW-3 and MSW-4, which exhibit the highest volatile organic compound (*V*_OC_) values, may be attributed to the strong donor moieties, such as the carbazole moiety in MSW-3 and triphenylamine in (MSW-4). Modification of the structure is important for significantly mitigating the charge recombination. This phenomenon contributed to the elevation of the (*V*_OC_) of MSW-3–4 and enhanced the overall efficacy of (DSSCs) reached to (0.610 and 0.651 eV). Among the four novel organic dyes, MSW-4 exhibited an efficiency reached to (6.27%) comparing to the reference D-5 dye that scored efficiency (5.93%) that mainly related to benzoxazolylacetonitrile (BOA) has a certain of electron-withdrawing ability due to character of both benzoxazole and cyano groups comparing to cyanoacetic acid that caused increasing in the values of *J*_SC_ increased from (12.68 to 13.60 mA cm^−2^) and *V*_OC_ increased from (0.632 to 0.651 eV). HOMO–LUMO simulations of the trimethoxy dye MSW-1 showed that the electrons were evenly spread across the molecule. This uniform distribution of MSW-1 is unfavorable for efficient charge separation because it hinders the effective movement of electrons towards the electrode. Consequently, this predicts the poor performance of MSW-1 in terms of photovoltaic efficiency compared to MSW-2–4 and D-5.

**Table 3:** Photovoltaic parameters of sensitizers MSW-1–4

Sensitizers (0.2 mM)	*J*_SC_ (mA cm^−2^)	*V*_OC_ (V)	FF (%)	*η*_cell_ (%)
D-5 dye	12.68	0.632	74.10	5.93
N719 dye	19.13	0.771	50.85	7.50
MSW-1	10.49	0.575	52.02	3.13
MSW-2	11.17	0.625	54.85	3.82
MSW-3	11.65	0.610	57.43	4.08
MSW-4	13.60	0.651	70.82	6.27
MSW-3 + MSW-4	20.07	0.721	55.14	7.98
MSW-3 + D-5	20.71	0.750	57.42	8.92
MSW-3 + N719	21.96	0.791	58.72	10.20

The effect of co-sensitization by using D-5, N719, and MSW-3 on the photovoltaic performance was studied. The open-circuit voltage (*V*_OC_), current density (*J*_SC_), efficiency (*η*), and fill factor (FF) of the fabricated DSSCs are listed in Table 3.

Through photovoltaic characterization of co-sensitized DSSCs, the goal of this study was to look at the relationship between the molecular structure of co-sensitizers MSW-4 with D-5, N719 ruthenium dye and MSW-3. Fig. 7b shows that the effectiveness of the produced cells is significantly influenced by the chemical structure of the co-sensitizers. The use of co-sensitizers may result in enhanced surface coverage owing to their small dimensions.^38^ However, when the carbazole co-sensitizer MSW-3 was co-sensitized with N719, the photovoltaic properties of N719 were much better. This enhancement is shown by the efficiency increase from (7.50 to 10.20%) for each individual case, the incorporation of co-sensitizers containing highly donating carbazole with a suitable size, enhanced the (*J*_SC_), that is shown to increase from 19.13 mA cm^−2^ to 21.96 mA cm^−2^. Co-sensitization of MSW-3 with the reference dye D-5, which has a cyanoacetic acid group as an anchoring moiety, enhanced the photovoltaic performance from 5.93% for D-5 to 8.92% for co-sensitization of (MSW-3 + D-5). This enhancement in efficiency may be attributed to an increase in the *J*_SC_ and *V*_OC_ values. Many studies have attributed the effect of MSW-3; the co-sensitization of MSW-4 with MSW-3 resulted in the lowest *J*_SC_ and PCE of all co-DSSCs, reaching 7.98%. The complementary light harvesting, optimal energetics, and kinetics facilitated by the dye cocktail (MSW-3 + N719) resulted in a synergistic improvement in all photovoltaic parameters. The co-sensitization strategy employs a cocktail of dyes with complementary absorption characteristics to achieve panchromatic light harvesting across the UV-visible spectrum.^39^ In this study, the reference N719 ruthenium dye displayed a typical metal-to-ligand charge transfer band at 540 nm with a limited spectral response. In contrast, the organic sensitizer MSW-3 contained donor–acceptor structures that extended absorption to shorter wavelengths through intramolecular charge transfer transitions. The complementary spectral coverage of (MSW-3 + N719) resulted in a higher IPCE in the blue-green wavelength range than that of N719 alone, which reached 87%. The addition of the D-5 dye to MSW-3, which has a donor–acceptor structure with a cyanoacrylic acid acceptor, further enhanced the IPCE compared to N719. As shown in Fig. 7c, the co-sensitized cells achieved excellent notable quantum efficiencies IPCE values of 66–87% over 350–530 nm owing to their high molar absorptivity, which generated notable quantum efficiencies. The IPCE order of MSW-3 + N719 > MSW-3 + D-5 > MSW-3 + MSW-4 > N719 matches the photocurrent trend (*J*_SC_).^40^ Optimized combinations of metal–complex N719 and organic dye MSW-3 enable synergistic enhancements in light harvesting, electron transfer, and charge transport for superior efficiency in co-sensitized solar cells.

### 4.1. Electrochemical characterization (EIS)

**Fig. 8:**
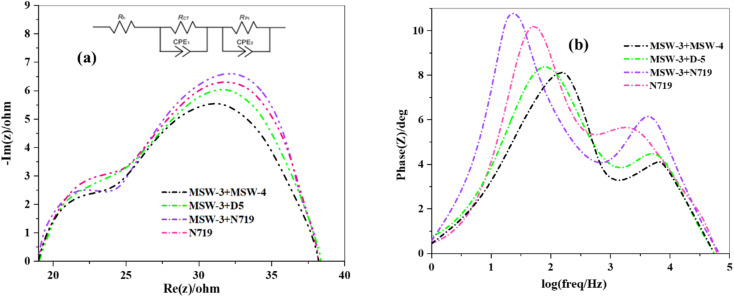
(a) Nyquist plots for co-sensitizers MSW-3, D-5, MSW-4 and N719, (b) Bode plots for co-sensitizers MSW-3, D-5, MSW-4 and N719.

Electrochemical impedance spectroscopy (EIS) was used to study the charge-transfer processes for (MSW-1–4) dyes.^41^ The semicircular represents the *R*_ct_ at the TiO_2_/dye/electrolyte interface. The *R*_ct_ values followed the order (MSW-3 + MSW-4) < (MSW-3 + D-5) < (N719) < (MSW-4 + N719) as shown in Fig. 8a. This matches the trend of (*V*_OC_) values. The co-sensitized devices (MSW-4 + N719) had a larger *R*_ct_ than N719 alone, indicating reduced electron recombination between the injected electrons and electrolyte. Co-sensitizing MSW-3 and D-5 decreased *R*_ct_ compared to MSW-3 sensitization alone but was higher than MSW-3 + MSW-4 co-sensitization. D-5 binds TiO_2_ strongly, facilitating electron injection, so a lower *R*_ct_ implies faster dye regeneration kinetics from better electronic coupling between MSW-3 and D-5. The MSW-3 + MSW-4 co-sensitized cell exhibited the smallest *R*_ct,_ indicating the lowest charge transfer resistance at the TiO_2_/dye/electrolyte interface. The Bode plots displayed in Fig. 8b are arranged in the following sequence (MSW-3 + MSW-4) < (MSW-3 + D-5) < (N719) < (MSW-4 + N719). Consequently, the higher *V*_OC_ values can be attributed to the longer recombination lifetime in the conduction band of TiO_2_. EIS revealed that co-sensitization with MSW-3 + N719 improved interfacial charge transfer owing to the facile dye regeneration of the Ru-complex N719 dye and the studied of stability of the co-sensitizers are approved in the (Fig. 14S) in the ESI.†

## 5 Conclusion

In summary, a set of novel organic sensitizers, specifically MSW-1–4, was successfully developed, with D–π–A configurations. The characteristics of these sensitizers, including their photophysical, electrochemical, and photovoltaic performance, were successfully manipulated by altering the link spacer and acceptor components. Computational computations employing DFT/TD-DFT yielded significant insights into the geometric and electrical characteristics of these dyes. Furthermore, one of the synthesized organic dyes, MSW-3, exhibited remarkable performance as a co-sensitizer when combined with the ruthenium complex (N719), reference dye D-5, and metal-free MSW-4. The co-sensitized cells exhibited significantly improved photoconversion efficiencies (PCEs), ranging from 7.89% to 10.20%, compared to those sensitized solely by N719 (7.50%). This enhancement can be attributed to the synergistic effect of improved (IPCE) and (*J*_SC_). Through IPCE and EIS analyses, it was observed that the incorporation of these dyes as co-sensitizers compensated for the photocurrent loss caused by the electrolyte, resulting in improved *J*_SC_. In conclusion, this study sheds light on the potential of highly efficient sensitizers of MSW-3 as a co-sensitizer for ruthenium-containing DSSCs. These findings provide valuable insights into the future development of more efficient and sustainable DSSCs.

## Consent to participate

All authors participated directly in the current research work.

## Consent to publish

The authors agree to publish the article under the Creative Commons Attribution License.

## Data availability

All relevant data are within the manuscript and available from the corresponding author upon request.

## Conflicts of interest

Authors declare that they have no conflict of interest.

## Supplementary Material

RA-014-D4RA04001E-s001
