# Supplementary material for: Unleashing synergistic co-sensitization of BOA dyes and Ru(ii) complexes for dye-sensitized solar cells: achieving remarkable efficiency exceeding 10% through comprehensive characterization, advanced modeling, and performance analysis
Source: RSC Adv. 2024 Aug 14;14(35):25549–60. doi: 10.1039/d4ra04001e (PMC11322857; doi:10.1039/d4ra04001e)
Supplement: RA-014-D4RA04001E-s001 [file RA-014-D4RA04001E-s001.pdf]

Supplementary Information (SI) for RSC Advances.
This journal is © The Royal Society of Chemistry 2024

Supporting Information

1. Materials and methods

Sigma-Aldrich and Alfa Aesar supplied the chemicals, which were utilized exactly as directed. JEOL's NMR spectrometer was used to record ^{1}H NMR and ^{13}C NMR spectra in DMSO-d_6 and $CDCl_3$ as solvents at 500 MHz. A Nicolet iS10 spectrometer from Thermo Scientific was used to produce Fourier transform IR spectra. UV-visible spectra were measured by using UV-Vis. spectrophotometer (T80 series). Thermo Scientific GC/MS model ISQ was used to record mass spectra. The Perkin Elmer 2400 analyzer was used to collect data for elemental analysis.

2. Spectral analysis:

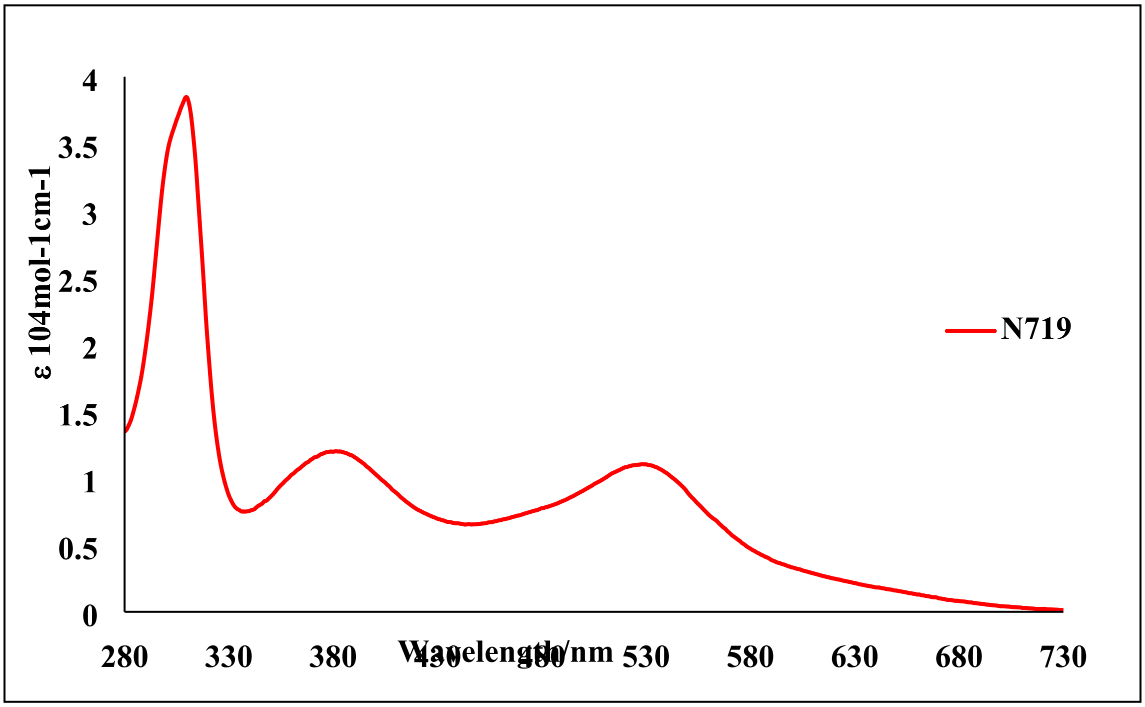

Fig. (S1): UV-Vis. absorption of N719

λ_{max} (nm)	ε ($10^4 M^{-1} cm^{-1}$)	λ_{onset} / nm	E_{0-0} (ev)
307, 384, 537	3.69, 1.18, 1.08	602	1.92

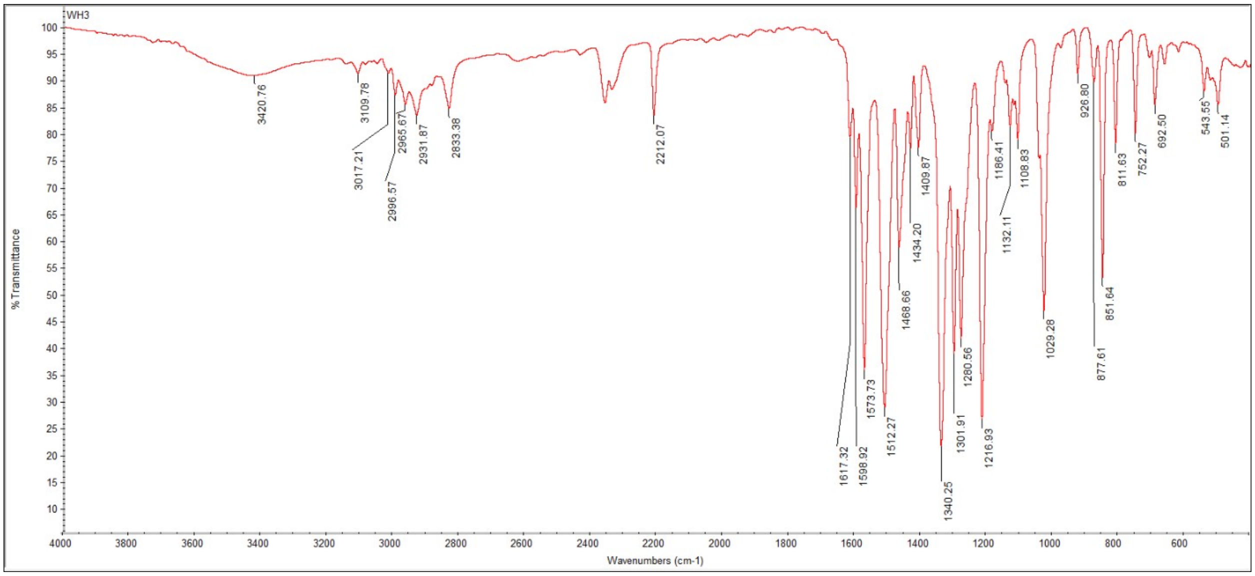

Figure (S2): ^{1}H NMR spectrum of MSW-1.

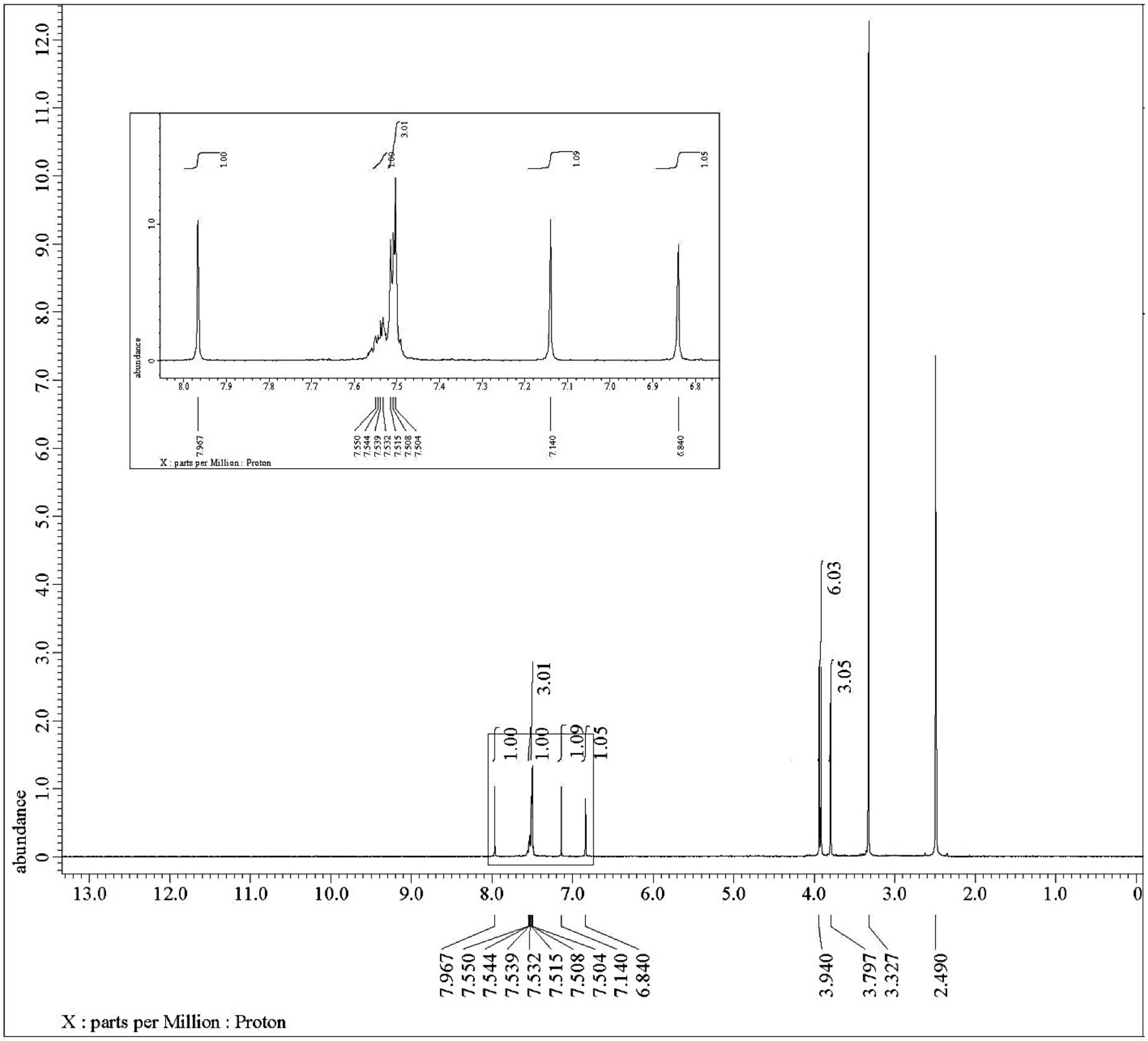

Figure (S3): ^{1}H NMR spectrum of MSW-1.

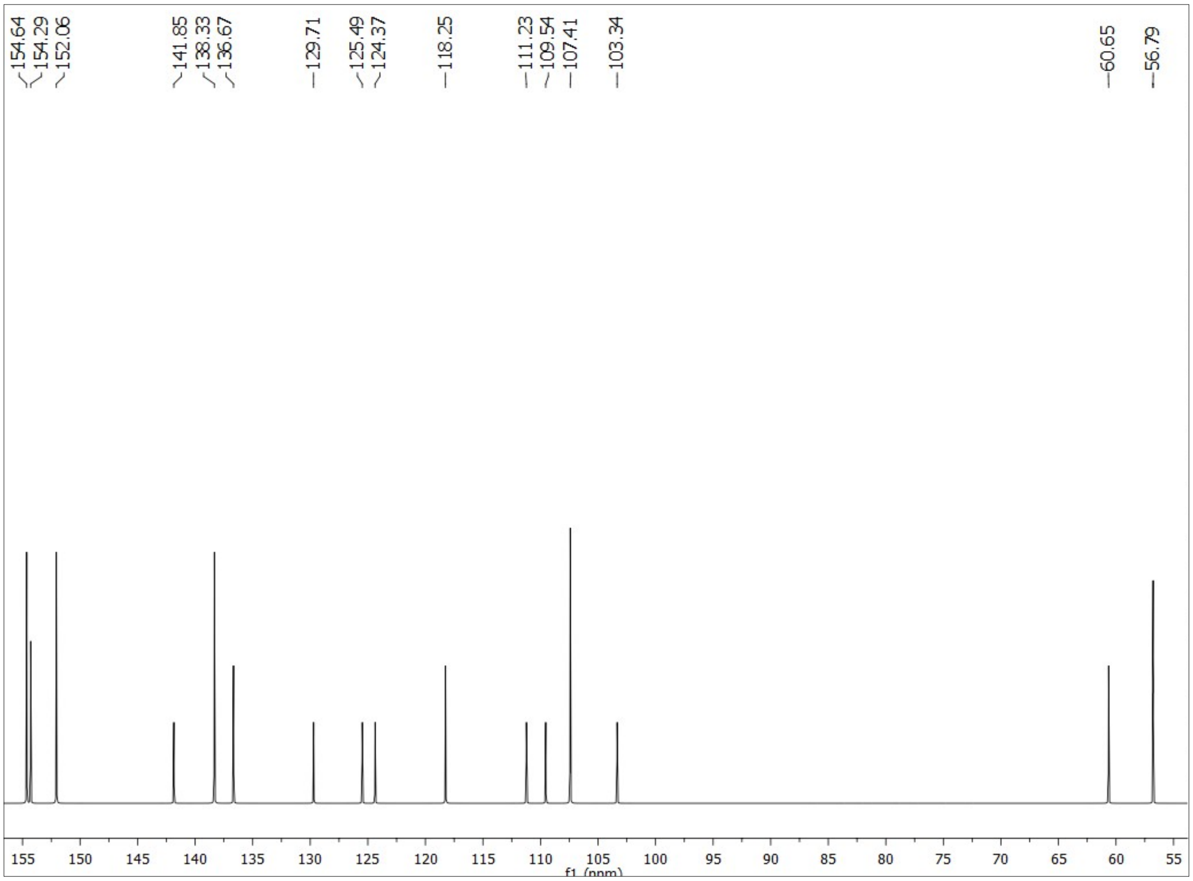

Figure (S4): ^{13}C NMR spectrum of MSW-1.

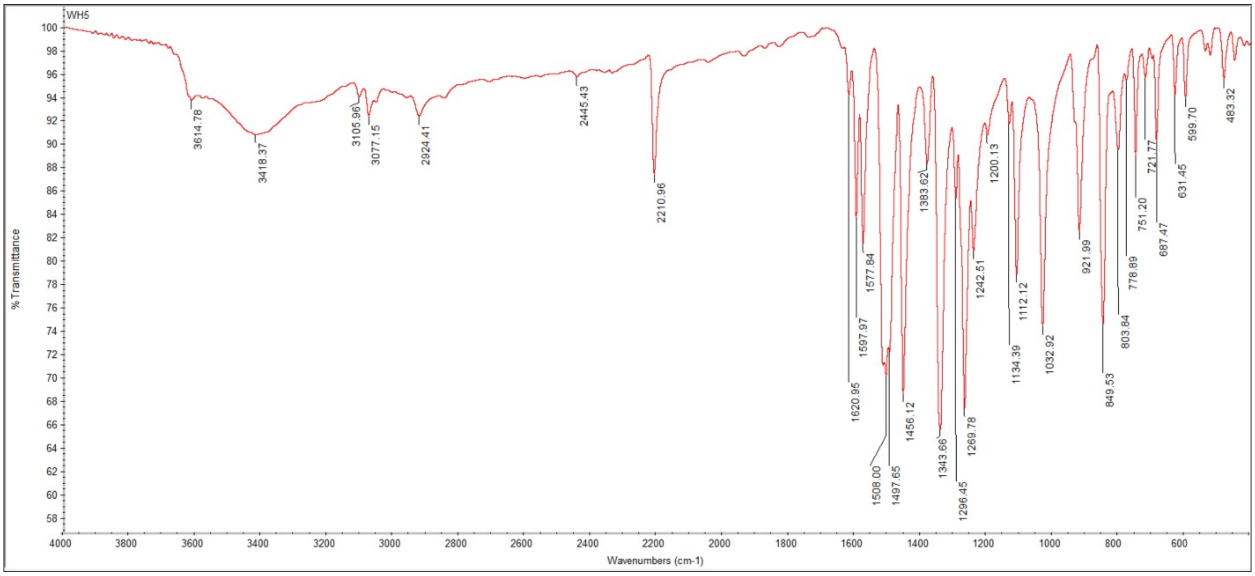

Figure (S5): IR spectrum of sensitizer MSW-2.

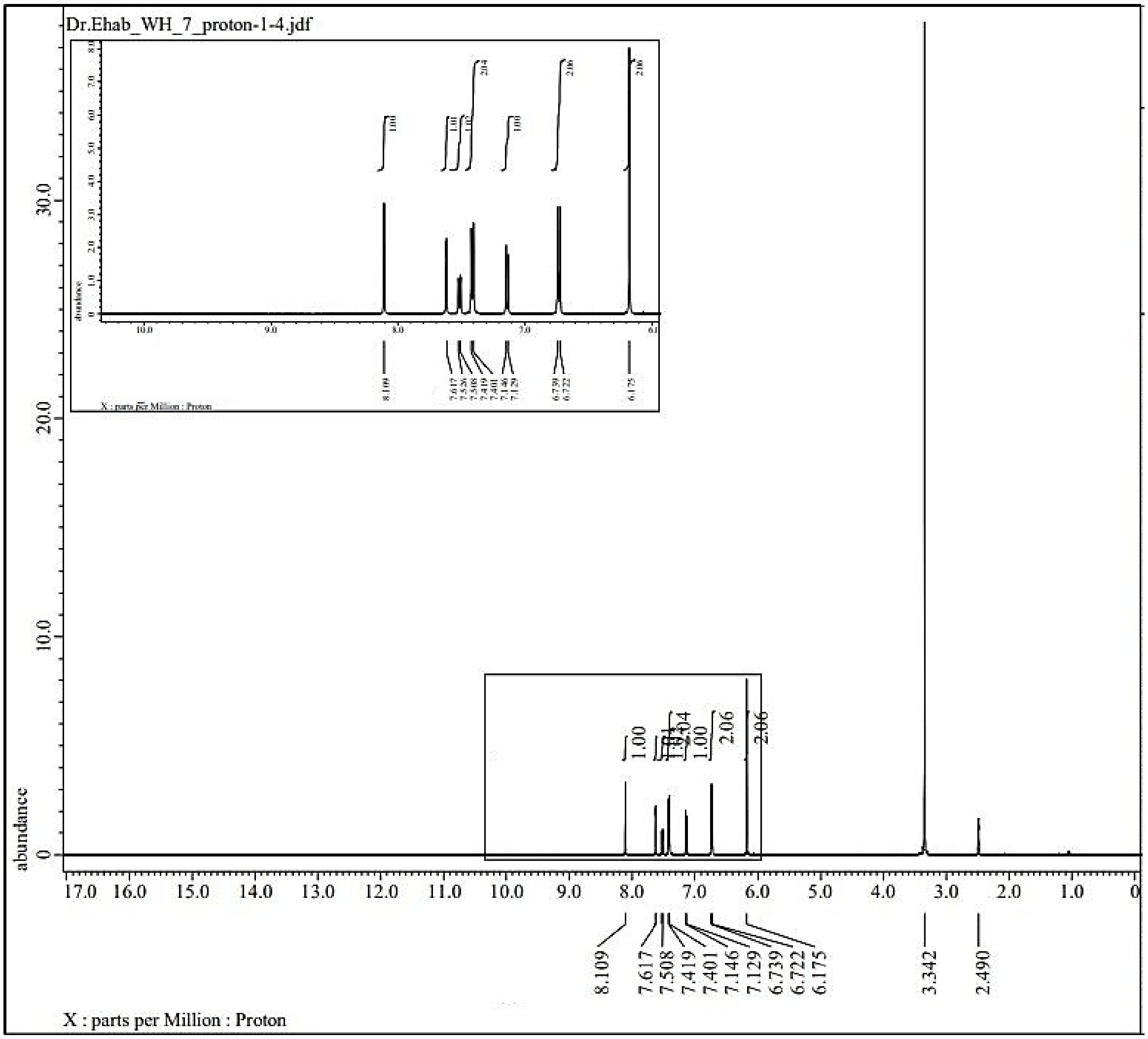

Figure (S6): ^{1}H NMR spectrum of sensitizer MSW-2.

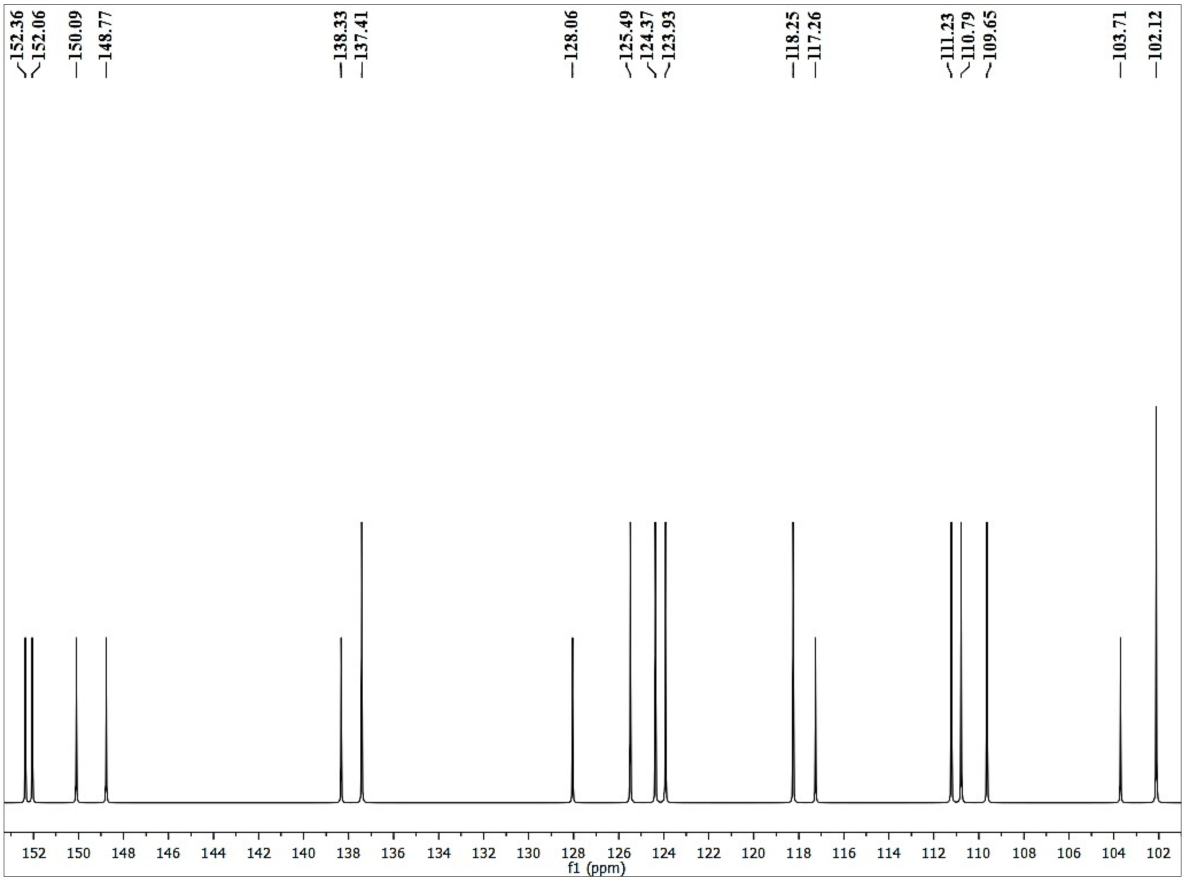

Figure (S7): ^{13}C NMR spectrum of sensitizer MSW-2

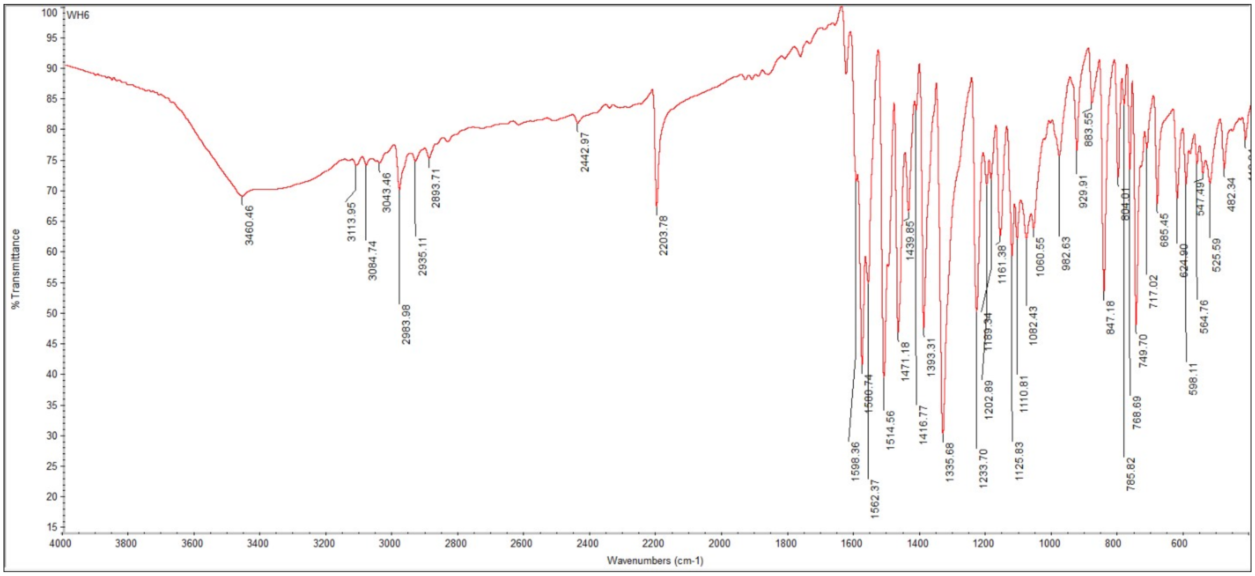

Figure (S8): IR spectrum of sensitizer MSW-3

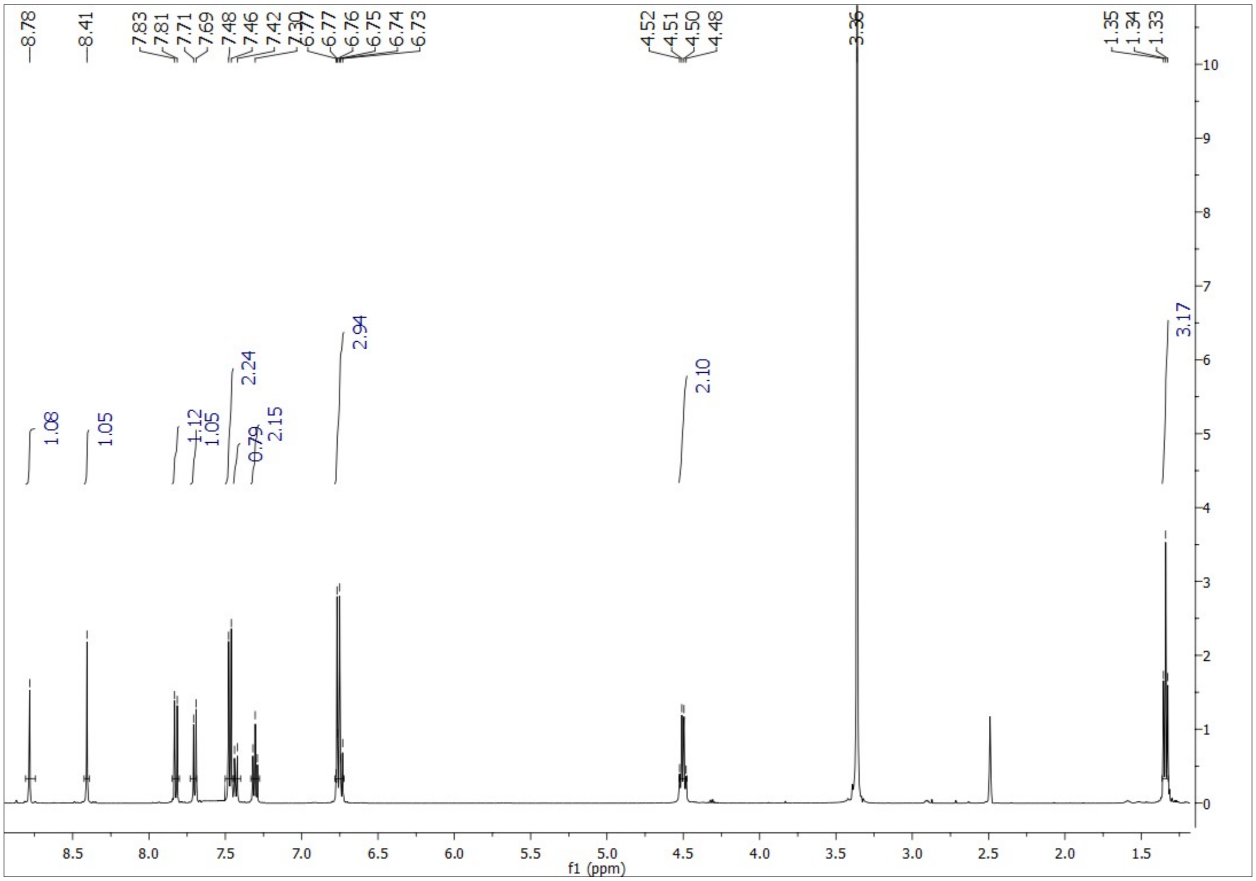

Figure (S9): ^{1}H NMR spectrum of sensitizer MSW-3.

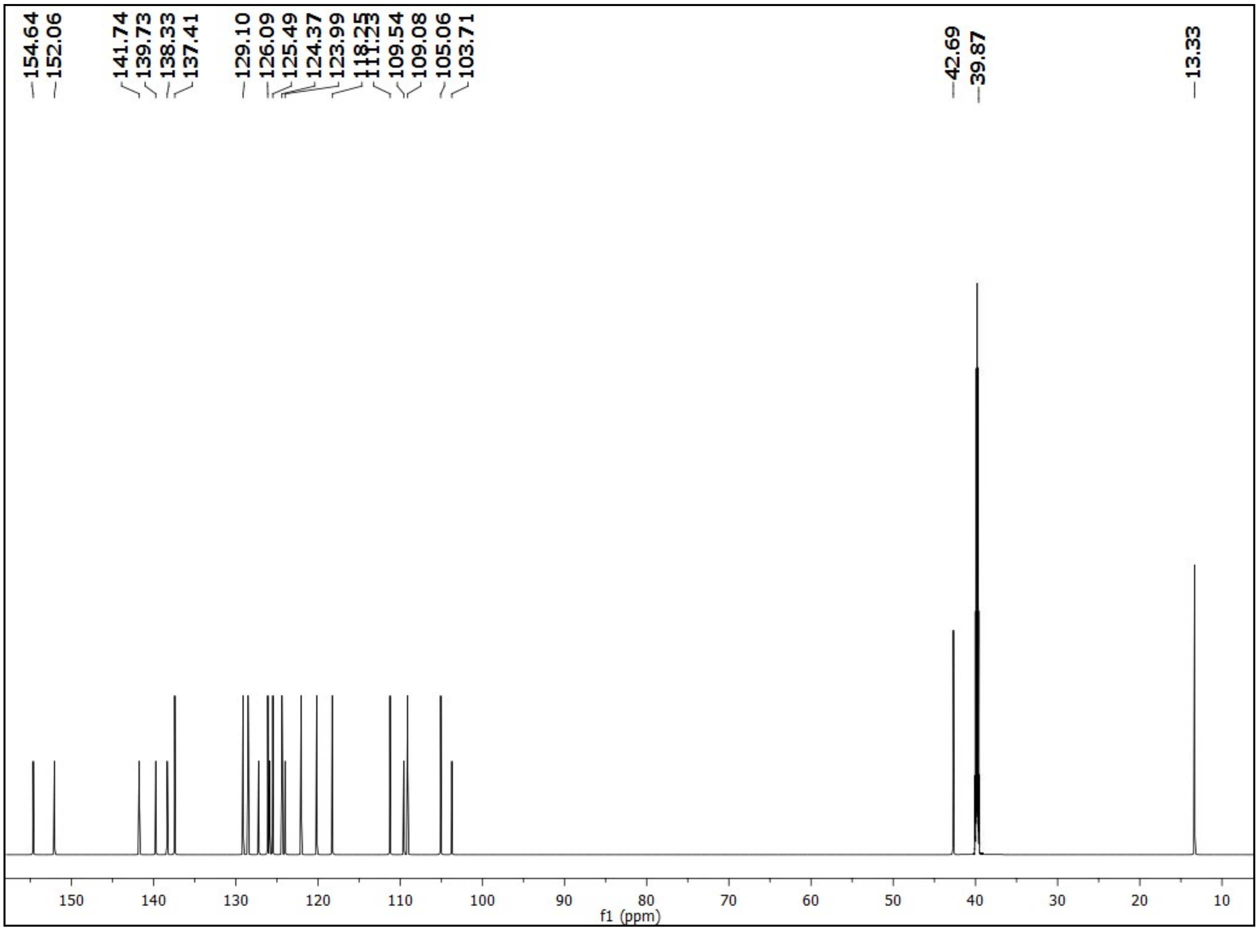

Figure (S10): C^{13} NMR spectrum of sensitizer MSW-3.

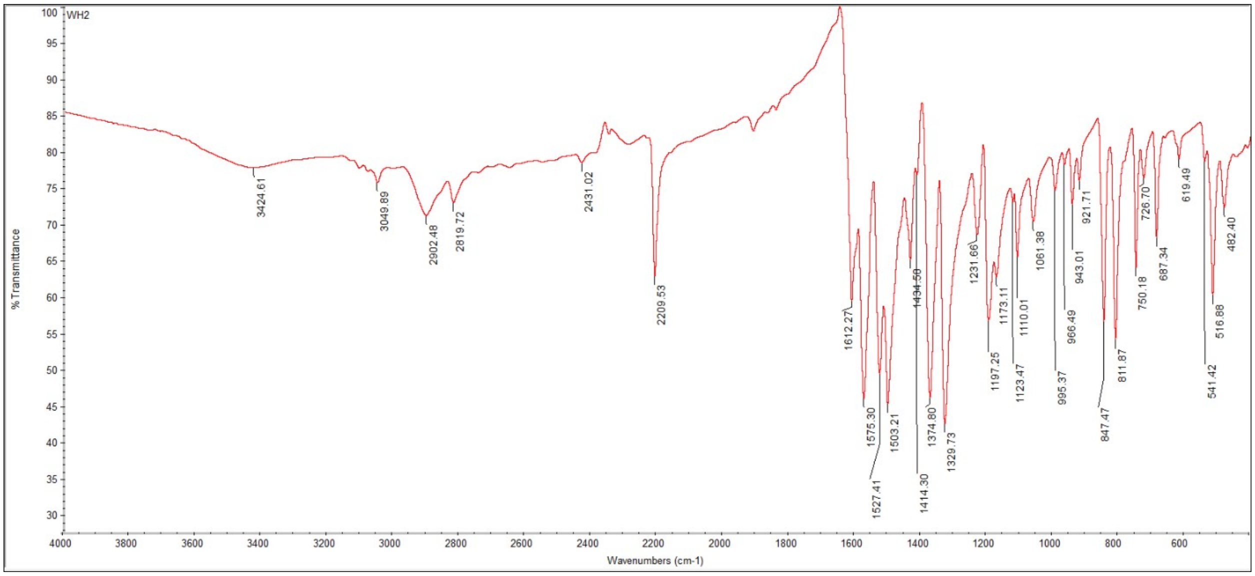

Figure (S11): IR spectrum of MSW-4.

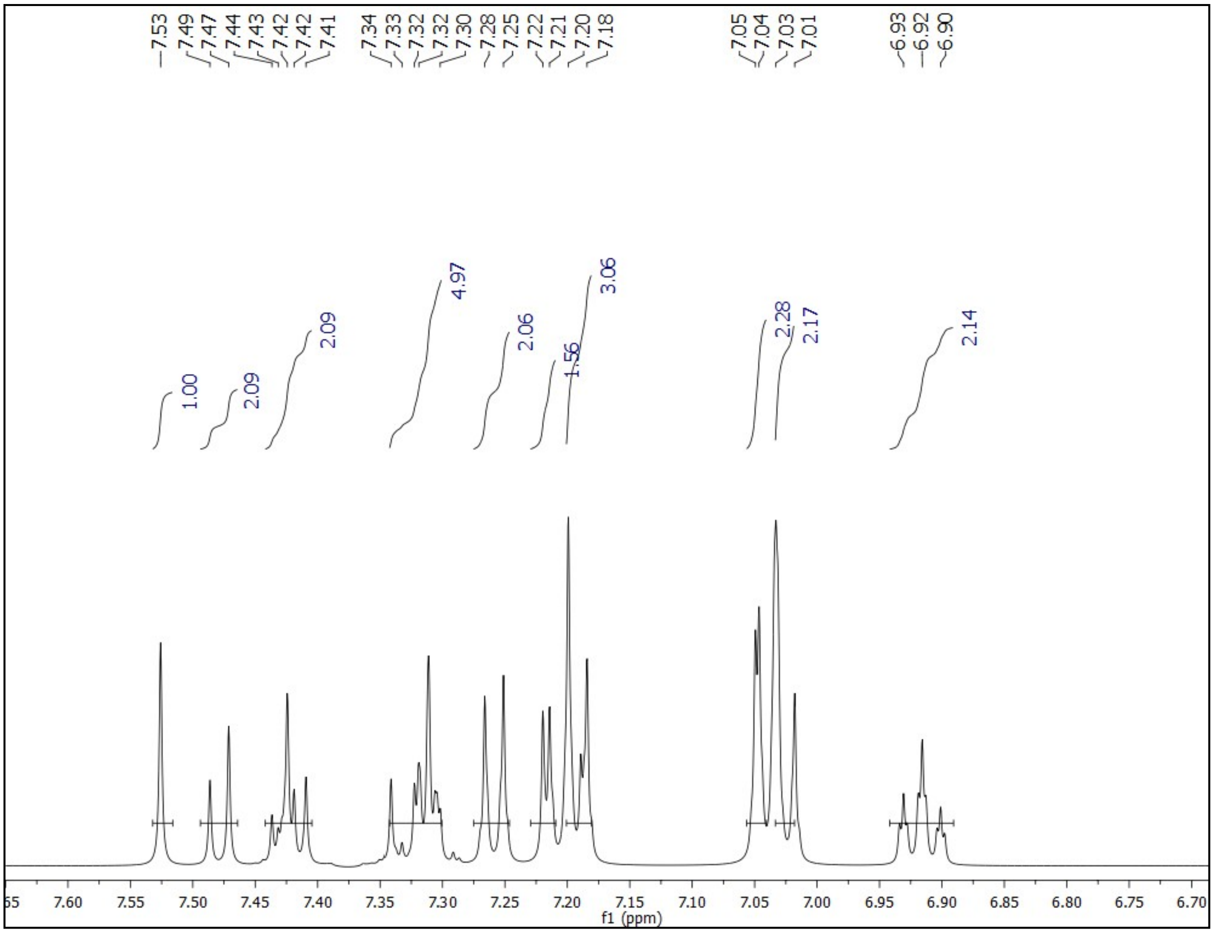

Figure (S12): ^{1}H NMR spectrum of MSW-4.

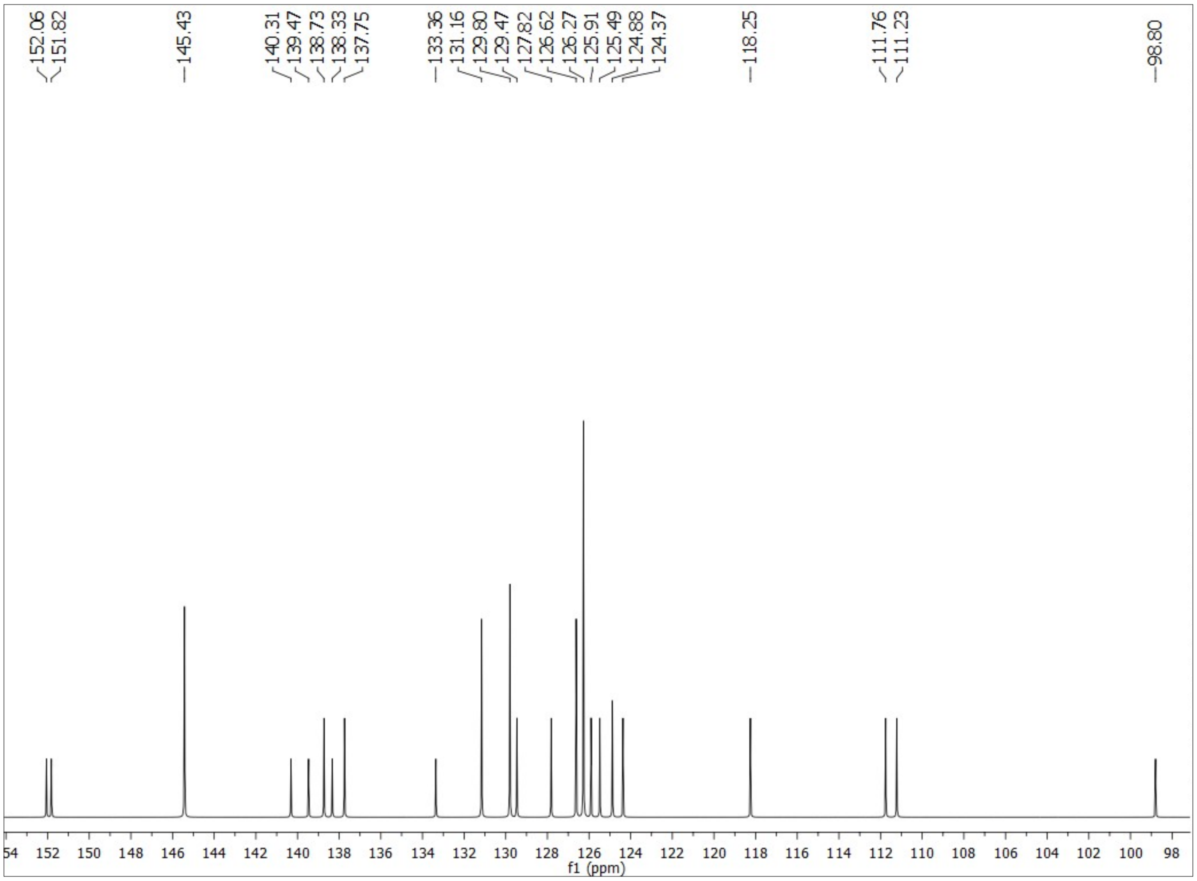

Figure (S13): ^{13}C NMR spectrum of MSW-4.

As shown in Fig. S14, the long-standing photostability of co-sensitizer of MSW-3 with N719, D-5 and **MSW-4** dye within DSSCs applications is clear from the sustained performance of the cell. Notably, even after 200 h of illumination, there was no discernible degradation in the initial performance. The excellent stability of sensitizers can be attributed to the inherent stability of the TPA **(MSW-4)** and ethyl carbazole (**MSW-3**) unit, which significantly contributes to its superior stability over time

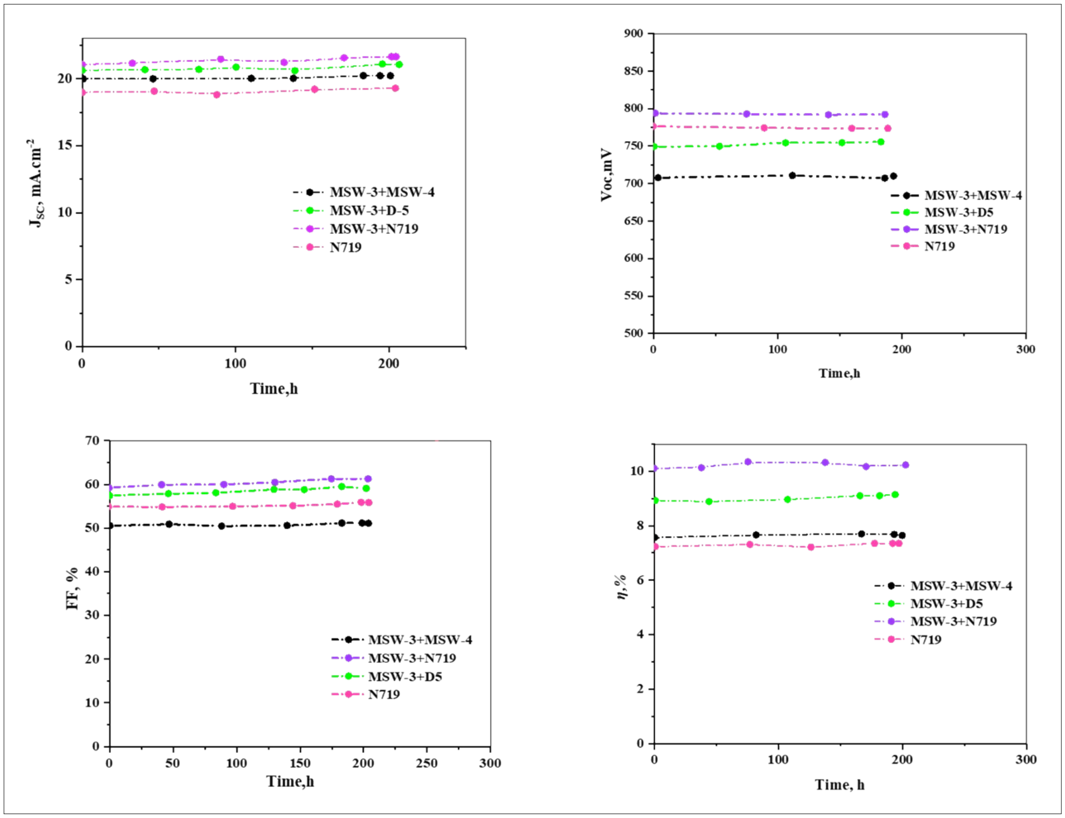

Fig. S14. Photovoltaic stability of the device of for co-sensitizers **MSW-3, D-5, MSW-4, and N719** measured under sunlight illumination for **2**00 h.

2. Cell preparations and photovoltaic characterizations

2.1. TiO_2 electrode preparation

A double-layer TiO_2 photoelectrode (10 + 5) µm in thickness with a 10 µm thick nanoporous layer and a 5 µm thick scattering layer (area: 0.25 cm^2) was prepared using a reported method. Fluorine-doped tin oxide (FTO) coated glasses (Nippon Sheet Glass Co., Japan) with a sheet resistance of 8-10 ohm-2 and an optical transmission of greater than 80% in the visible range were screen printed using anatase TiO_2 colloids (particle size ~ 13 nm) obtained from commercial sources (Ti-Nanoxide D/SP, Solaronix). Nanocrystalline TiO_2 thin films were deposited onto the conducting glass by screen-printing, which was then sintered at 500 °C for 1 h. The film thickness was

measured with a Surfcom 1400A surface profiler (Tokyo Seimitsu Co. Ltd.). The electrodes were impregnated with a 0.05 M titanium tetrachloride solution and sintered at 500 °C. The films were further treated with 0.1 M HCl(aq) before examination. The dye solutions of the main sensitizers **MSW-1-4** were prepared in 1:1:1 mixture of acetonitrile, tert-butyl alcohol and dimethyl sulfoxide (DMSO).

For Co-sensitization we used two different concentration of **MSW-3** (0.2 mM) with the 0.2 mM **N719, D-5** and **MSW-4** and followed the same processing as above. The electrodes were immersed in the dye solutions and then kept at 25 ° C for 20 h to adsorb the dye onto the TiO_2 surface. For preparing the counter electrode, pre-cut TCO glasses were washed with water followed by 0.1M HCl in EtOH, and sonication in acetone bath for 10 min. These washed TCO were then dried at 400 °C for 15 min. Thin layer of Pt-paste (Solaronix, Platisol T/SP) on TCO was printed and the printed electrodes were then cured at 450 °C for 10 min.

2.2. Fabrication of dye-sensitized solar cell

Photovoltaic and incident photon-to-current efficiency (IPCE) measurements were made on sandwich cells, which were prepared using TiO_2 coated working electrodes and platinum coated counter electrodes and were sealed using a 40 μm Syrlyn spacer through heating of the polymer frame. The redox electrolyte (Solaronix, Iodolyte HI-30) consisted of a solution of 0.6 M DMPII, 0.05 M I_2, 0.1 M LiI and 0.5 M TBP in acetonitrile.

2.3. Photovoltaic measurements

Photovoltaic measurements of sealed cells were made by illuminating the cell through the conducting glass from the anode side with a solar simulator (WXS-155S-10) at AM 1.5 illuminations (light intensity: 100 mW cm^{-2}).

2.4. Incident photon to current efficiency (IPCE) conversion

IPCE measurements were made on a CEP-2000 system (Bunkoh-Keiki Co. Ltd.). IPCE at each wavelength was calculated using Equation 1, where I_{SC} is the short-circuit photocurrent density (mA. cm^{-2}) under monochromatic irradiation, q is the elementary charge, λ is the wavelength of incident radiation in nm and P0 is the incident radiative flux in W/m^2.

$$IPCE(\lambda) = 1240\left(\frac{I_{SC}}{q\lambda P_o}\right) \qquad (1)$$

2.5. Electrochemical impedance spectroscopy (EIS)

The electrochemical impedance spectra were measured with an impedance analyzer potentiostat (Bio-Logic SP-150) under illumination using a solar simulator (SOL3A, Oriel) equipped with a

450 W xenon lamp (91160, Oriel). EIS spectra were recorded over a frequency range of 100 mHz to 200 kHz at room temperature. The applied bias voltage was set at the V_{OC} of the DSSCs, with AC amplitude set at 10 mV. The electrical impedance spectra were fitted using Z-Fit software (Bio-Logic).

2.6. Cyclic voltammetry

Cyclic voltammetry (CV) was performed in DMF with the electrolyte 0.1 M $[TBA][PF_6]$ at a scan rate of 50 mV s^{-1}. The working electrode used is the Glassy carbon, Pt wire represented the counter electrode and the reference electrode is Ag/Ag^+ in ACN. Fc/Fc^+ was introduced as internal reference.

4. Molecular Modeling

Equilibrium molecular geometries of AH-1-5 calculated using the Becke's three parameter hybrid functional, Lee–Yang–Parr's gradient corrected correlation functional (B3LYP) and (6-311g(d, p)) [1, 2, 3, 4]. The geometry optimization calculations were followed by energy calculations using time-dependent density functional theory (TD-DFT) utilizing the energy, functional B3lyp and the basis set 6-311g (d, p). The solvent (DMF) effect was accounted for by using the conductor-like polarizable continuum model (C-PCM), implemented in Gaussian 09.

References

[1] G. Melikian, F. Rouessac, C. Alexandre, Synth Commun. 1993, 23, 2631

[2] A. D. Becke, Phys. Rev. A 1988, 38, 3098.

[3]C. T. Lee, W.T. Yang, R.G. Parr, Phys. Rev. B. 1988, 37, 785

[4] N. Godbout, D.R. Salahub, J. Andzelm, E. Wimmer, Optimization of Gaussian-type basis-sets for local spin-density functional calculations .1. Boron through neon, optimization technique and validation. Can. J. Chem.-Rev. Can. Chim. 1992, 70, 560-571.
